# The effect of DNA-binding proteins on insertion sequence element transposition upstream of the *bgl* operon in *Escherichia coli*

**DOI:** 10.3389/fmicb.2024.1388522

**Published:** 2024-04-11

**Authors:** Peter W. Kopkowski, Zhongge Zhang, Milton H. Saier

**Affiliations:** Department of Molecular Biology, School of Biological Sciences, University of California, San Diego, La Jolla, CA, United States

**Keywords:** IS element, insertional mutation, adaptive mutation, Crp, IHF, DNA-binding protein

## Abstract

The *bglGFB* operon in *Escherichia coli* K-12 strain BW25113, encoding the proteins necessary for the uptake and metabolism of β-glucosides, is normally not expressed. Insertion of either IS1 or IS5 upstream of the *bgl* promoter activates expression of the operon only when the cell is starving in the presence of a β-glucoside, drastically increasing transcription and allowing the cell to survive and grow using this carbon source. Details surrounding the exact mechanism and regulation of the IS insertional event remain unclear. In this work, the role of several DNA-binding proteins in how they affect the rate of insertion upstream of *bgl* are examined via mutation assays and protocols measuring transcription. Both Crp and IHF exert a positive effect on insertional Bgl^+^ mutations when present, active, and functional in the cell. Our results characterize IHF’s effect in conjunction with other mutations, show that IHF’s effect on IS insertion into *bgl* also affects other operons, and indicate that it may exert its effect by binding to and altering the DNA conformation of IS1 and IS5 in their native locations, rather than by directly influencing transposase gene expression. In contrast, the cAMP-CRP complex acts directly upon the *bgl* operon by binding upstream of the promoter, presumably altering local DNA into a conformation that enhances IS insertion.

## 1 Introduction

Since their discovery, transposable elements (“jumping genes”) have been studied in prokaryotic and eukaryotic models. In both types of organisms, they are best known for their ability to insert into variable locations within an organism’s genome (McClintock, 1950). In some cases, transposition takes place upstream of or within a structural gene, which may cause a change in protein expression for the former and loss of gene function for the latter (Whiteway et al., 1998; Zhang and Saier, 2009a,b).

One of the best characterized examples of this phenomenon is IS (Insertion Sequence) element insertion into the *bglGFB* operon, which is not expressed in wild-type (WT) *Escherichia coli* K-12 strain BW25113. Both the pathogenic and non-pathogenic forms of *E. coli* contain this operon, and to our knowledge it is never expressed in WT. Binding of the global histone-like nucleoid structuring (H-NS) protein at two sites on either side of the otherwise active *bgl* promoter is the most important factor in silencing transcription of the *bgl* operon by a strong repression mechanism (Schnetz, 1995; Dole et al., 2004; Lam et al., 2022). Data published by other groups indicate that the upstream and downstream H-NS binding sites exhibit synergy with each other (Nagarajavel et al., 2007). Building upon this discovery, recent data from our group suggest the formation of a repression loop structure that blocks access of RNA polymerase to the *bgl* promoter (Lam et al., 2022; Tran et al., 2022).

**Figure 1:**
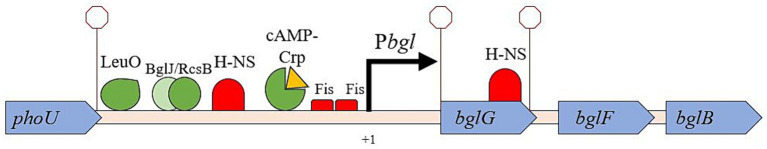
The *bgl* operon with relevant DNA-binding protein sites and structural features. The *phoU*/*bglG* intergenic region is where IS insertion occurs, upstream of the promoter. Several DNA binding proteins are known to bind to the *bglGFB* regulatory region and influence transcription. A major focus of this work is to determine whether protein binding in this region affects the rate of insertion of IS1 and/or IS5.

The first gene in the operon, *bglG*, contains the downstream H-NS binding site within its coding region and is itself flanked by two Rho-independent terminators, limiting the amount of RNA transcript made of *bglG* as well as the two other downstream genes, *bglF* and *bglB* (Figure 1; Mahadevan and Wright, 1987; Schnetz and Rak, 1988). BglG is a homodimer that binds to its own transcript to prevent early termination, allowing transcription to continue and thereby promoting expression of the entire operon (Amster-Choder and Wright, 1992). BglG also has other functions including the positive regulation of insertional and non-insertional Bgl^+^ mutations, although how it accomplishes these functions has yet to be elucidated (Zhang et al., 2022). The *bglF* gene immediately follows *bglG*’s downstream terminator and encodes a membrane-integrated protein responsible for the uptake and concomitant phosphorylation of β-glucosides via a phosphotransferase (PTS)-dependent mechanism (Fox and Wilson, 1968). Thus, BglF passes a phosphoryl group from HPr or a BglG monomer to the incoming β-glucoside concomitant with transport (Chen et al., 1997), marking it for hydrolysis of the aglycone from the glucose-phosphate moiety by BglB, the operon’s third and final gene product (Prasad et al., 1973), and this sugar-P feeds directly into glycolysis. Since phosphorylated BglG is in equilibrium with phosphorylated BglF, transfer of the phosphoryl group from BglG to BglF causes the dephosphorylation of BglG, allowing it to dimerize into its active anti-termination configuration. BglG can be phosphorylated on two specific histidyl residues, one that promotes antitermination and the other which prevents antitermination (Görke and Rak, 1999; Rothe et al., 2012).

While repression of *bgl* operon expression by H-NS is strong, preventing almost 100% of the maximal transcription rate, transposition of an IS element may occur upstream of the *bgl* promoter (Reynolds et al., 1981). The vast majority of IS elements that insert into this area are either IS1 or IS5, and both elements insert in either orientation (forward or backward) within an area spanning ~200 bp (Reynolds et al., 1981). This insertional event eliminates repression of *bgl* operon expression by H-NS (Lopilato and Wright, 1990; Singh et al., 1995), increases *bgl* operon expression several hundred-fold (Lam et al., 2022), and allows cells to grow, divide and form colonies using β-glucosides as their sole carbon source (Prasad and Schaefler, 1974). This state corresponds to a “Bgl^+^” phenotype.

Two details about this insertional event remain of great interest. First, Bgl^+^ mutations of any class, insertional or otherwise, only occur when *E. coli* cells are starving in the presence of a β-glucoside, which can be freely taken in and used after activation of the *bgl* operon via mutation or IS insertion. Second, the rate of insertion into *bgl* under these circumstances is much higher than the random mutation rate for *E. coli* (Hall, 1998).

Since mutations are widely considered by the scientific community to be random events rather than environmentally directed, the above facts indicate that the *bgl* operon is operating under an additional, fundamentally novel layer of regulation that is not well understood. Several paradigms like *bgl* exist, where insertional events occurring under specific circumstances of cellular stress lead to a phenotype which relieves that stress. These operons deserve further study so that the mechanisms of their regulation may be incorporated into today’s accepted models of mutation and evolution.

As noted above, *bgl* is one of several operons in *E. coli* that are preferential sites for insertion of IS elements. These sites are commonly found in the operon’s promoter region, have high Gibbs free energy signatures, and exhibit an increase in IS insertion frequency when the bacterial cell is experiencing a specific type of stress related to the operon’s function (Humayun et al., 2017). These so-called superhelical stress-induced duplex destabilization (SIDD) sites are under study by our group and others to determine whether insertion into a SIDD site is directed by environmental conditions, as well as to uncover whether SIDDS evolved specifically to allow IS-mediated operon activation (Humayun et al., 2017).

Since the hydrogen bonds between individual base pairs are relatively weak in a SIDD site, the opening of the DNA may be what allows IS insertion to occur there with increased frequency. This would support previous unpublished findings that H-NS lowers the rate of insertion into the *bgl* operon (Z.Z., unpublished data). When H-NS binds at or near the SIDD site, it may prevent it from opening to a conformation that allows for easy insertion of an IS element. It is therefore important to identify what roles, if any, that DNA-binding proteins have upon transposition into the sequences on or near their binding sites. In pursuit of this goal, we studied several DNA-binding proteins to better understand their roles in regulating the insertion of IS elements and the phenomena of “adaptive” or “directed” mutations in general. Adding to the body of knowledge of how transposons and their movements are regulated is vital for proper understanding of the “mobilomes” that are present in almost every life form on Earth.

Integration Host Factor (IHF) is a heterodimeric global histone-like DNA-binding protein involved in several cellular functions including transcriptional regulation, DNA recombination, and chromosome compaction (Goosen and van de Putte, 1995; Engelhorn and Geiselmann, 1998). After binding to the minor groove of the double helix, it significantly bends the DNA at least 160° per dimer (Sugimura and Crothers, 2006). Under starvation conditions, IHF concentrations rise 4–8-fold in *E. coli* (Bushman et al., 1985; Ditto et al., 1994) and has been implicated in the induction of proteins related to carbon starvation (Sclavi et al., 2007). Also of interest is IHF’s role in the transposition of Mu prophage in *Pseudomonas putida*. *In vitro* studies of IHF binding to the Mu promoter suggest that IHF assists in formation of a transposase complex that may facilitate excision via supercoiling relief (Surette et al., 1989; Allison and Chaconas, 1992). *In vivo*, IHF is not required for Mu transposition, but has a dual effect on Mu transposase transcription. IHF binds to the end of the Mu transposase promoter region and activated transcription of the transposase while simultaneously relieving repression by H-NS at a downstream site (van Ulsen et al., 1996). Multiple groups have shown that a Gly to Glu mutation at the 62nd amino acid in IhfA causes loss of IHF’s ability to bind to its DNA consensus sequence but does not prevent dimerization with IhfB (Granston and Nash, 1993; Hales et al., 1994).

Crp is a DNA-binding protein that relies upon cyclic AMP (cAMP) to become activated (Kolb et al., 1993). It is a global transcriptional regulator that affects expression of almost 200 genes in *E. coli* (Fic et al., 2009). Most applicable to this work, cAMP-Crp is generally involved in the positive regulation of genes concerned with the metabolism of carbon sources (Deutscher, 2008; Görke and Stülke, 2008) and is a necessary factor in the activation of *bgl*. Even if repression by H-NS is relieved, *bgl* remains transcriptionally inactive and unable to grow using only β-glucosides, unless activated Crp is present (Mukerji and Mahadevan, 1997; Gulati and Mahadevan, 2000).

In this work, we provide evidence that two DNA-binding proteins affect the rate of IS insertion into *bgl*: the cAMP-Crp complex and Integration Host Factor (IHF). Both proteins are positive regulators of insertion; that is, their presences and abundances maintain or increase the frequencies of IS-related Bgl^+^ mutations observed in WT cells. The effect of IHF is not specific to *bgl* but applies to several other operons into which IS elements insert in response to environmental stresses. Our results suggest that binding of IHF to known sites in IS1 and IS5 does not influence transposase gene transcription, and is therefore likely to influence DNA conformation, transpososome formation and/or energetics at the site of transposition.

## 2 Results

### 2.1 Deletion of *ihfA* decreases the Bgl^+^ mutation rate

**Figure 2:**
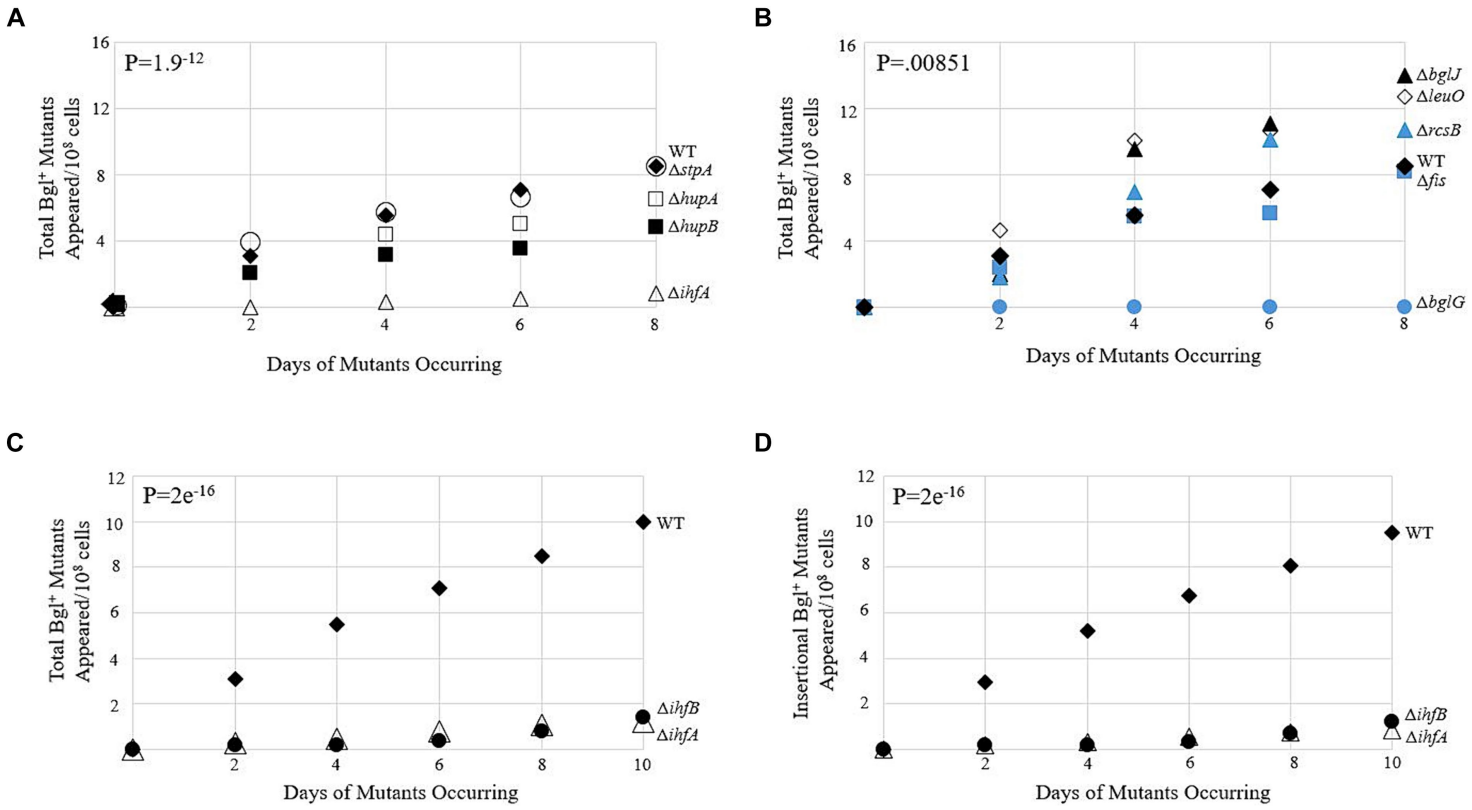
Bgl^+^ mutation frequencies due to deletion of genes encoding DNA-binding proteins. **(A,B)** Appearances of total Bgl^+^ mutants over time. Mutation assays were carried out on M9 + 0.5% salicin plates as described in Materials and Methods. Strains are loosely grouped in A and B based on their global nature **(A)** or more specific binding to *bgl.* **(B,C)** Effects of deleting *ihfA* and *ihfB* on appearance of total Bgl^+^ mutants. **(D)** Effects of deleting *ihfA* and *ihfB* on appearance of insertional Bgl^+^ mutants. Colony PCR using primers flanking the region of *bgl* insertion was performed to differentiate between insertional and non-insertional colonies. WT = ♦; Δ*ihfA* = ∆; Δ*bglG* = ●; Δ*stpA* = ○; Δ*hupA =* □; Δ*hupB* = ■; Δ*bglJ* = ▲; Δ*leuO* = ◊; Δ*rcsB =* ▲; Δ*fis* = ■; Δ*ihfB =* ●.

The first phase of our research was to determine which genes, if any, demonstrated a clear effect on the frequency of IS insertion into *bgl* upon deletion. Figures 2A,B show data gathered from several single deletion strains over a 10-day period. The full list of deletion strains tested is tabulated in Supplementary Table S1. Genes were selected for study based on the DNA-binding abilities of their gene products and were loosely separated into two groups: the global histone-like DNA-binding proteins and operon-specific DNA-binding proteins with known binding sites within the *bgl* control region. Colony PCR was performed on the mutant colonies of each strain, using primers that flank the P*bgl* regulatory region into which IS1 and IS5 are known to insert (Supplementary Table S2). Of the deletion strains tested, ∆*ihfA* was selected for further study, since it caused the greatest change in the frequency of Bgl^+^ mutant appearances compared to WT (Figure 2A). Further confirmatory Bgl^+^ mutation assays were performed on WT and ∆*ihfA* to precisely ascertain the difference in mutant incidence between them. These results again showed an approximately eight-fold drop in total Bgl^+^ mutants in ∆*ihfA*, and a more than 11-fold drop in insertional mutants (Figures 2C,D). Deletion of *ihfB*, encoding IHF’s other subunit IhfB, showed the same effect as ∆*ihfA* (Figures 2C,D). To determine whether the ∆*ihfA* mutation prevented cell multiplication but not necessarily insertion, IS5 was inserted upstream of *bgl* in its usual position in the ∆*ihfA* mutant. This strain grew as quickly on salicin media as WT Bgl^+^ cells. This indicates that ∆*ihfA* acts specifically to lower the IS insertion frequency rather than hinder the growth of Bgl^+^ mutants after insertion takes place. These observations led us to consider the mechanism of IHF’s role in IS insertion, as well as its specificity for the *bgl* operon.

### 2.2 Further characterization of an *ihfA* deletion mutant

**Figure 3:**
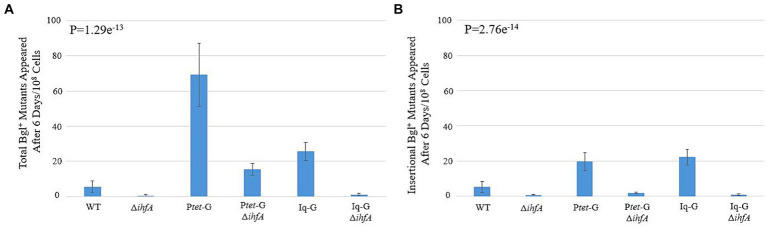
Effect of *ihfA* deletion combined with increased *bglG* expression on Bgl^+^ mutations. **(A)** Effect of deleting *ihfA* on total Bgl^+^ mutations in the cells over expressing *bglG*. **(B)** Effect of deleting *ihfA* on insertional Bgl^+^ mutations in the cells over expressing *bglG*. Bgl^+^ mutation assays were performed as previously described. Two *bglG* overexpression strains, P*tet*-G (P*tet* driving *bglG* at the *intS* locus) and Iq-G (*lacIq* driving *bglG* at the *intS* locus), were used together with or without the *ihfA* deletion. This experiment serves to determine if the *ihfA* deletion has similar inhibitory impact on IS1/IS5 insertions in the cells with higher levels of BglG. Our previous study showed that more BglG produced leads to greater insertional frequencies (Zhang et al., 2022). Colony PCR was used to differentiate insertional and non-insertional Bgl^+^ mutants.

The ∆*ihfA* background was transferred to the backgrounds P*tet*-G and Iq-G, which constitutively express *bglG* at the intS locus, leaving the native *bgl* operon intact. Our objective was to observe how IHF’s observed effect would interact with increased BglG levels, which has been previously shown to increase the rate of Bgl^+^ insertional and non-insertional mutations (Zhang et al., 2022). The results presented in Figures 3A,B show that the stimulatory effect of increased *bglG* expression on insertion frequency counteracts ∆*ihfA’s* negative effect on the same. As with the WT background, deletion of *ihfA* in either background led to a greater than 10-fold drop in insertional mutants. In the case of P*tet*-G, the total mutants fell by four-fold when *ihfA* was deleted, and a more than 10-fold decrease was observed among insertional mutants. These data further solidify BglG’s effect on insertional mutation rate and confirm our results on *ihfA* deletion, which appears to mitigate both the insertional and non-insertional mutant incidences in the P*tet*-G strain, but not the Iq-G strain.

### 2.3 Characterization of P*tet-*driven *ihfA/B* expression

Since the IHF protein has a significantly positive effect on IS insertion upstream of the *bgl* promoter, we decided to determine if changing the amounts of both IhfA and IhfB subunits would have a different effect. PK01, a strain expressing both the *ihfA* and *ihfB* genes in the chromosome using the constitutive P*tet* promoter, was therefore constructed and subjected to a mutation assay as previously described. Interestingly, a decrease in both total and insertional mutants was observed after 10 days (Supplementary Figures S1A,B). We consider several possible reasons for why this occurred (see Discussion).

PK01_R was constructed, containing the *tetR* gene. *tetR* encodes the P*tet* repressor protein TetR, which is most effective in the absence of its inducer, any one of several tetracycline derivatives. Our goal was to see if different levels of P*tet* induction would reveal a similar trend as that already observed with normal PK01. Supplementary Figures S1C,D show the results of a mutation assay as described, but with the addition of several different concentrations of the *Tet* inducer anhydro-tetracycline (aTC). As expected, the increase of IhfA and IhfB led to more Bgl^+^ mutations. The number of mutants that appeared was like the previous assay at levels of maximal aTC induction (~30 μM), supporting the results of Supplementary Figure S1B.

**Figure 4:**
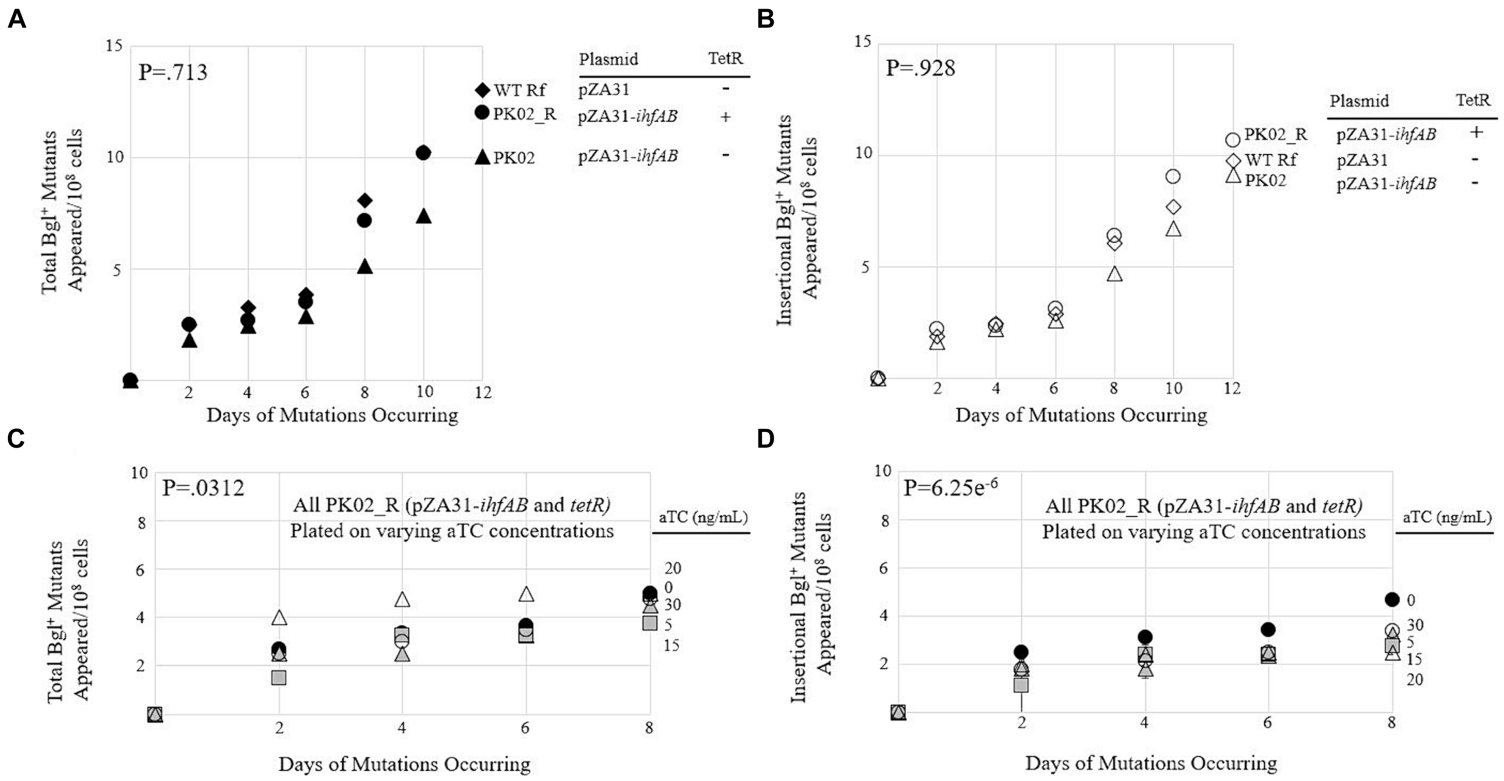
Overexpression of *ihfA* and *ihfB* on a plasmid slightly lowers the IS insertional rate into *bgl*. **(A)** Effect of IhfA and IhfB overproduction on total Bgl^+^ mutations. **(B)** Effect of IhfA and IhfB overproduction on insertional Bgl^+^ mutations. **(C)** Effect of titrating *ihfA* and *ihfB* expressions on a plasmid on total Bgl^+^ mutations. **(D)** Effect of titrating *ihfA* and *ihfB* expressions on a plasmid on insertional Bgl^+^ mutations. For **(C,D)**, mutation assays were performed as previously described on M9 salicin plates containing aTC at 0 to 30 ng/mL over a 10-day period. 0aTC = ●; 5aTC = ▲; 15aTC = ■; 20aTC = ∆; 30aTC = ○.

To create a strain expressing both IHF subunits at even higher levels, a pZA31 plasmid containing P*tet*-driven *ihfA* and *ihfB* was constructed and then transformed into wild type BW25113 and its counterpart containing the *Tet* repressor gene *tetR*. These new strains, called PK02 and PK02_R, respectively, were compared to each other and an isogenic control (WT Rf) in a mutation assay. All three strains behaved very similarly (Figures 4A,B), suggesting that the increased transcription of both IHF subunits did little to influence the insertion rate. To confirm this, PK02_R was plated on M9 salicin media containing differential levels of the Tet promoter’s inducer aTC (Figures 4C,D). The amount of inducer present did not appear to cause a corresponding change in total or insertional Bgl^+^ mutants, as consistent with the findings in Figures 4A,B.

### 2.4 Changing IHF levels has no significant effect on transcription of *bgl*

**Figure 5:**
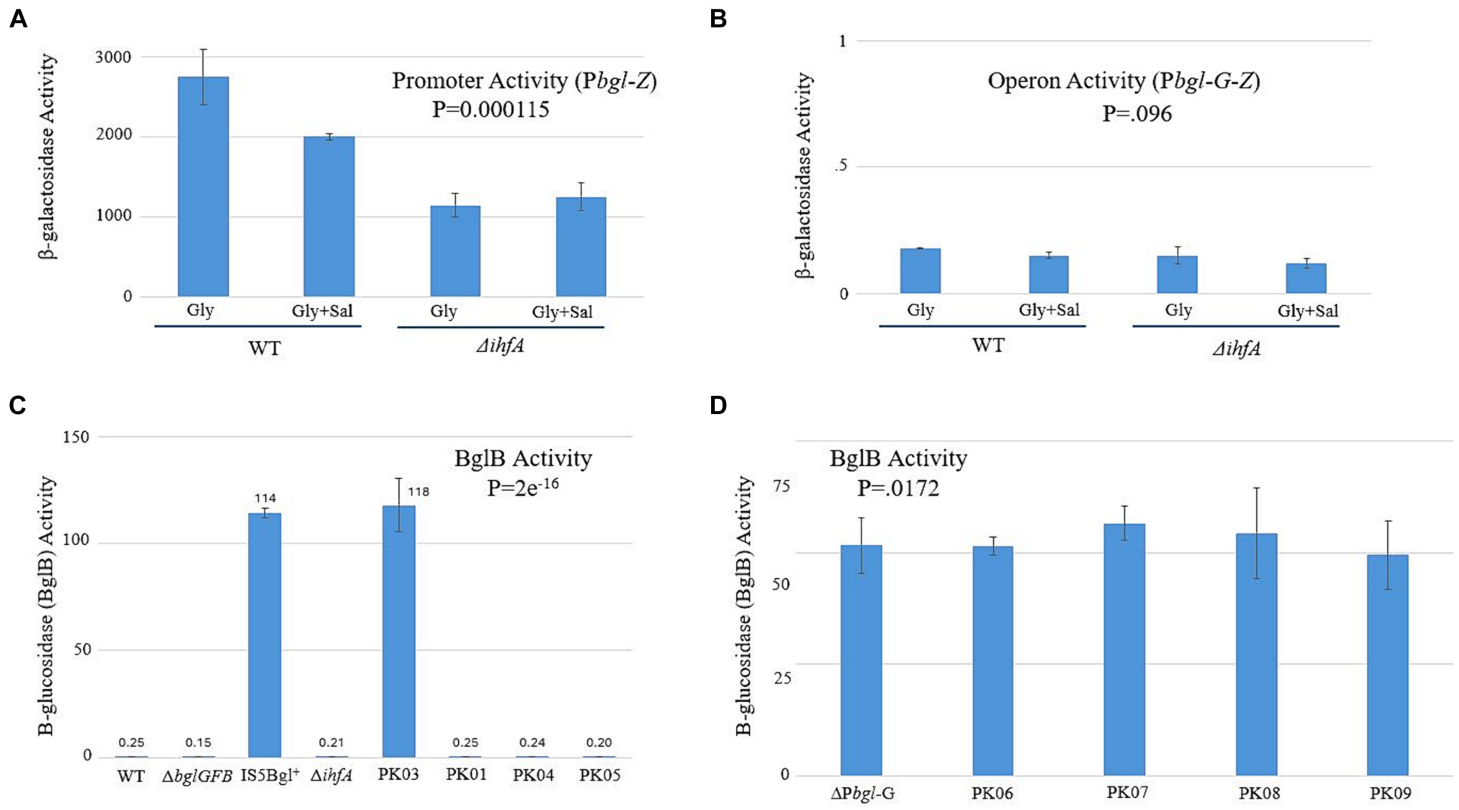
Changing IHF levels does not have a significant effect on *bgl* operon transcriptional activity. LacZ and BglB assays were carried out as previously described (Materials and Methods). **(A)** The *bglGFB* promoter (P*bgl*) activities determined using the LacZ assay. **(B)** The *bglGFB* operon (P*bgl*-*bglG*) activities determined using LacZ assay. **(C)** The *bglGFB* operon activities were determined via the BglB assay. PK01 = P*tet*-*ihfA*_P*tet*-*ihfB*; PK03 = ∆*ihfA* IS+; PK04 = ∆*ihfA* in G50; PK05 = P*tet*-*ihfA*_P*tet*-*ihfB* in G50. **(D)** The *bglGFB* operon activities in strain ∆P*bgl*-G determined using the BglB assay. Strain ∆P*bgl*-G was deleted for the *bglG* gene plus its flanking terminators and is Bgl^+^. PK06 = ∆*ihfA* in ∆P*bgl*-G; PK07 = ∆*fis* in ∆P*bgl*-G; PK08 = ∆*ihfA* and ∆*fis* in ∆P*bgl*-G; PK09 = P*tet*-*ihfA*_P*tet*-*ihfB* in ∆P*bgl*-G.

Our next goal was to establish a mechanism by which IHF exerts its effect on the expression of *bgl*. If ∆*ihfA*’s effect on the frequency of Bgl^+^ insertional mutations is due to the inability of IHF binding to the *bgl* upstream region, then LacZ assays could show a difference in transcriptional activity when IHF is present versus when it is absent. The constructs we devised for determining this are shown in Supplementary Figure S2. The native *bgl* operon was left intact to maintain normal induction by β-glucosides. A second, altered *bgl* promoter and its upstream region were positioned in front of the native *lacZ* gene in place of its usual promoter. This new construct, P*bgl-Z*, can measure the promoter activity of this second *bgl* operon. A similar construct, P*bgl-G-Z*, contained the *bglG* gene between P*bgl* and *lacZ* for the purpose of determining operon activity. Figures 5A,B show the *lacZ* activity results, which demonstrated a ~ 2-fold change in promoter activity but no significant change in operon activity between WT and ∆*ihfA.* We repeated these experiments using a BglB assay, which functions similarly in practice to the LacZ assay. However, this assay uses PNP-Glucoside (PNPG) as its substrate and measures activity of the *bgl* operon directly, rather than having to rely on additional constructs and the *lacZ* gene. The results of the BglB assay confirmed those of the LacZ experiments, showing no increase or decrease in *bgl* activity (Figure 5C). We suspected that any potential change may not be detectable due to the natural low levels of *bgl* activity; to investigate this possibility, the ∆P*bgl*-G strains were constructed. ∆P*bgl*-G strains are similar to the strains originally used for BglB but exhibit a 200-fold increase in operonic activity due to the removal of the *bglG* gene, both of its flanking terminator sequences, and the downstream H-NS binding site. To see if ∆P*bgl*-G would lead to observable differences between strains, BglB assays were run again using several previously used strains now in a ∆P*bgl*-G background. No large change was observed across the board, leading us to reject the notion that specific binding of IHF significantly affects transcription of *bgl* (Figure 5D).

### 2.5 The effects of *ihfA* deletion on expression of several IS-activated operons

**Figure 6:**
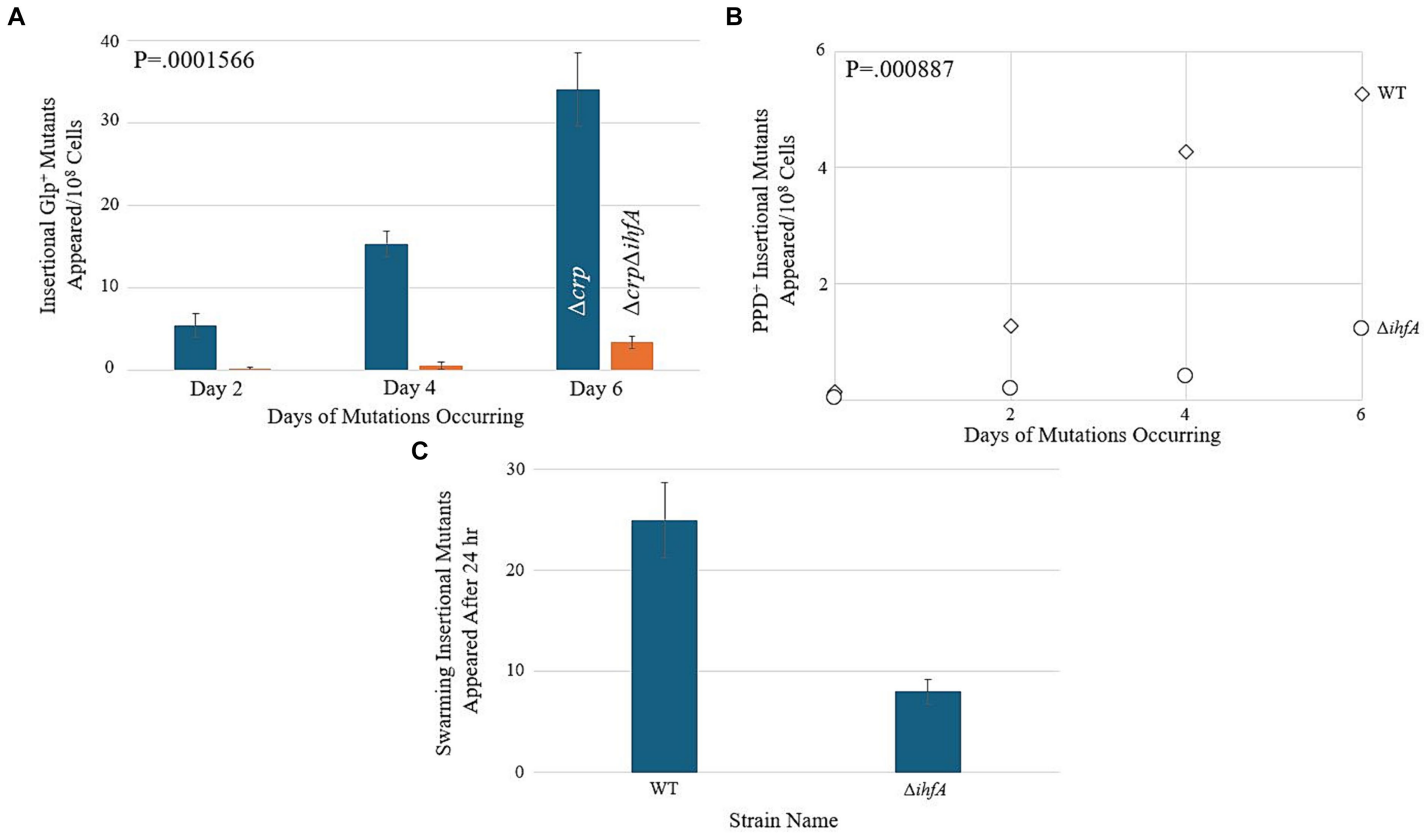
Deletion of *ihfA* affects IS element insertion into other operons. **(A)** Effect of *ihfA* deletion on Glp^+^ mutations. A ∆*crp* mutant and a ∆*crp* ∆*ihfA* double mutant were subjected to the Glp^+^ mutation assay on M9 + 0.5% glycerol media as previously described. **(B)** Effect of *ihfA* deletion on PPD^+^ mutations. WT and ∆*ihfA* strains were subjected to the PPD^+^ mutation assay on M9 + 1% propanediol media. **(C)** Effect of *ihfA* deletion on swarming mutations. WT and ∆*ihfA* were subjected to the swarming mutation assay on 0.3% LB agar media.

Since our LacZ data did not reveal a substantial change in transcriptional activity within the context of *bgl*, we hypothesized that ∆*ihfA* does not affect *bgl* directly but may exert its effect on insertion upstream of *bgl* by a nonspecific means. If IHF, being nonspecific, is important for IS insertion in general, then it may be involved in the upstream process of IS excision from other locations in the genome. In this situation, other operons into which IS1 and IS5 insert would also experience a change in insertion frequency upon deletion of *ihfA*. To explore this possibility, a ∆*ihfA* strain was tested alongside an isogenic WT strain in mutation assays corresponding to several other operons where preferential IS insertion into their respective SIDD sites have been previously documented (McCalla et al., 1978; Chen et al., 1989; Whiteway et al., 1998; Zhang et al., 2010, 2017; Humayun et al., 2017). Agar plates were made with the intention of promoting insertion into the *glpFK*, *fucAO*, and *flhDC* operons, and were inoculated with a number of cells specific to the paradigm tested. The results for these three operons are presented in Figures 6A–C, and all three showed a significant decrease in insertional frequency, just as observed for *bgl*.

As when testing *bgl*, these experiments compared ∆*ihfA* to the WT strain. However, the parameters of each experiment differed depending on the specific operon being tested. Interestingly, all the operons examined showed a significant decrease in the insertional mutant appearance rate of the ∆*ihfA* counterpart, suggesting that the dependency of this process on IHF affects several operons into which insertions occur, and consequently, it may affect an early step (IS excision or transposition) of the IS1 or IS5 element itself.

### 2.6 Examining the mechanism of the non-specific effect of IHF on IS1/IS5 transposition via loss of *ihfA*’s DNA-binding function and LacZ measurements of IS1/IS5 promoters

**Figure 7:**
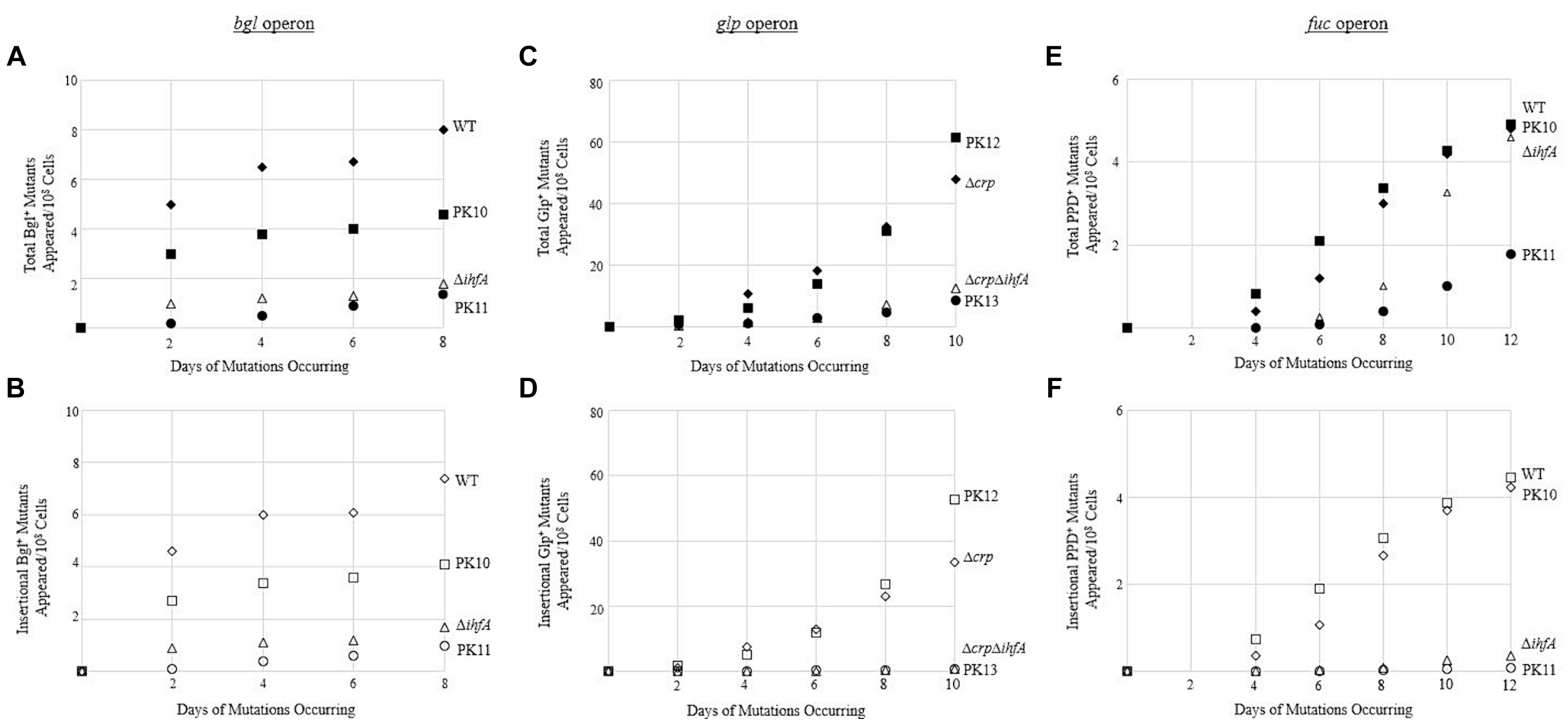
Binding of IHF is necessary to effectively exert its positive effect on IS insertion upstream of the *bgl* operon. **(A,B)** Negative effect of expressing *ihfA*_G62E on total Bgl^+^ mutations **(A)** or on insertional Bgl^+^ mutations **(B)**. **(C,D)** Negative effect of expressing *ihfA*_G62E on total Glp^+^ mutations **(C)** or on insertional Glp^+^ mutations **(D)**. **(E,F)** Negative effect of expressing *ihfA*_G62E on total PPD^+^ mutations **(E)** or on insertional PPD^+^ mutations **(F)**. IhfA with the G62E mutation loses the DNA binding property but is still able to dimerize with IhfB. The dimers consisting of IhfA_G62E and IhfB are incapable of DNA binding. WT and Δ*crp* = ♦ total and ◊ insertional; PK10 and PK12 = ■ total and □ insertional; Δ*ihfA* and Δ*crp*Δ*ihfA* = ▲ total and ∆ insertional; PK11 and PK13 = ● total and ○ insertional.

Since our view of IHF’s effect proved to be relevant to other operons, the question remained: What is the mechanism of the IHF effect on transposition? First, we tested whether the DNA-binding ability of IHF was the cause of the observed multi-operon effect. A mutation assay was performed comparing WT and ∆*ihfA* to two other strains. These strains have P*tet*-driven *ihfA* at the *intS* locus, but here the *ihfA* gene product contains a G-E substitution at the 62nd position (referred to as G62E). The product of this *ihfA* mutant loses its DNA-binding function but is still able to dimerize with IhfB (Granston and Nash, 1993; Hales et al., 1994). The *ihfA_*G62E construct was placed into WT as well as the deletion strain for *ihfA* at its native position (∆*ihfA*), yielding PK10 and PK11. If binding to DNA was important for IHF’s effect, then PK11 would show similar insertion rates as ∆*ihfA,* while PK10 would have binding and nonbinding IhfA and therefore would not experience as drastic of a change. Figures 7A,B show the results of a *bgl* mutation assay, conducted as previously described. The strains acted according to expectations, with PK10 having fewer mutants than WT, but more than its ∆*ihfA* counterpart PK11.

To confirm these results, the G62E constructs were also used in mutation assays for the *glpFK* and *fucAO* operons as previously described; the results of these are in Figures 7C–F. The G62E strains act similarly with respect to other operons as with *bgl*; in all operons tested, the G62E mutant generated around the same number of mutations as the WT if the native *ihfA* gene was intact. However, the G62E mutation in the ∆*ihfA* deletion mutant still caused a drastic drop to the same level of ∆*ihfA* or lower.

### 2.7 IHF has no observable effect on transcription of the transposase gene encoded within IS1 or IS5

**Figure 8:**
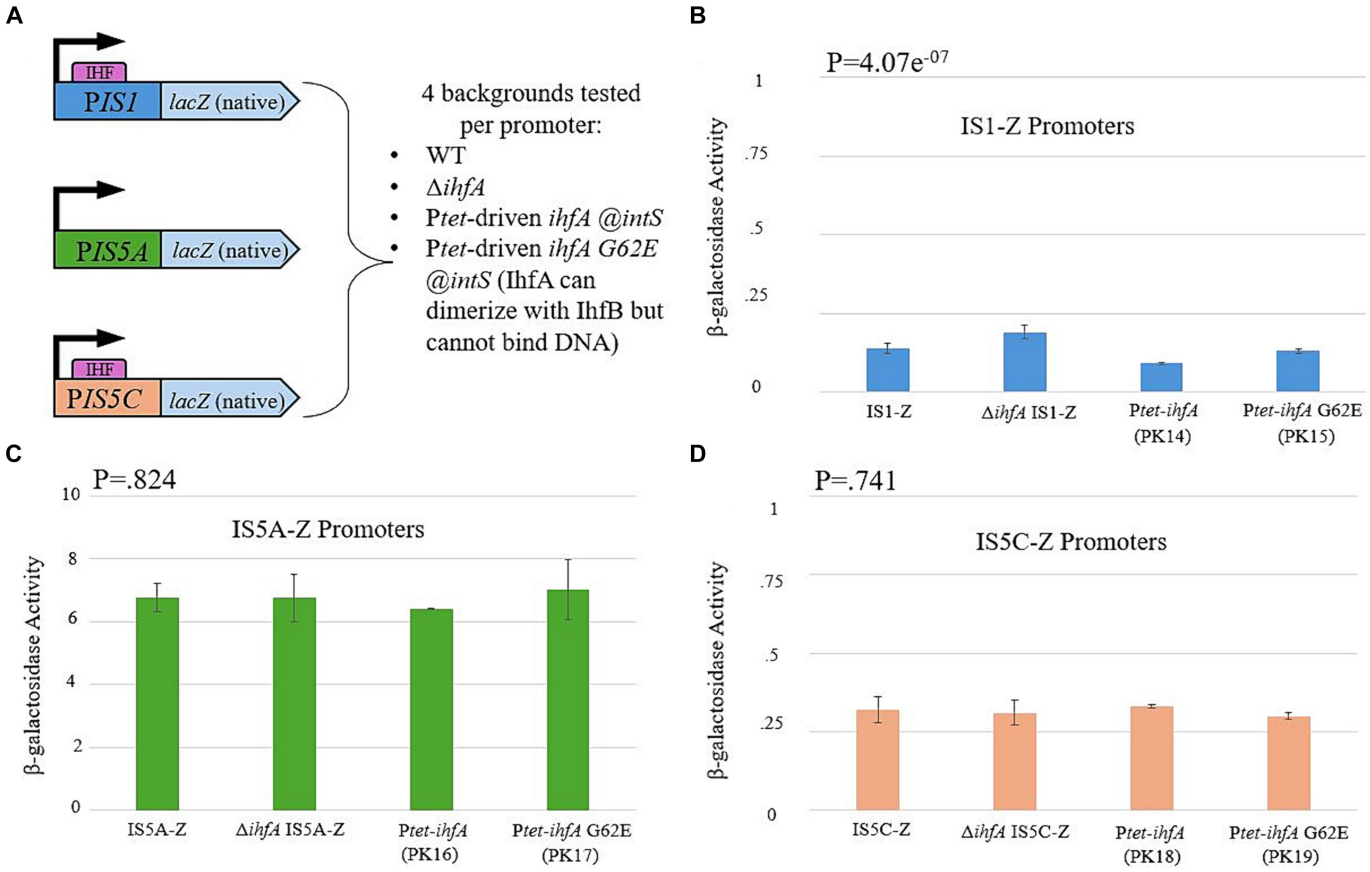
IHF does not influence transcriptional activity of IS1 or IS5. **(A)** A graph showing the construction of each LacZ reporter, which uses the promoter region of the 5′ end of IS1 and both ends of IS5 (PIS5A and PIS5C). **(B)** The IS1 transposase promoter (PIS1) activity in various genetic backgrounds. **(C)** The *ins5A* transposase promoter (PIS5A) activity in various genetic backgrounds. **(D)** The *ins5BC* operon promoter (PIS5C) activity in various genetic backgrounds.

Research conducted by other laboratory groups had demonstrated that IHF binding sites are present at both ends of IS1 and on one end of IS5 (hereafter referred to as IS5C; IS5A is the end without an IHF binding site) (Gamas et al., 1987; Prentki et al., 1987; Muramatsu et al., 1988). We therefore decided to test whether the presence and/or binding of IHF causes a change in the level of transcription of either or both IS elements. This was accomplished by placing the promoter regions for IS1 and both ends of IS5 (containing the Inverted Repeats to which IHF binds) directly before the native *lacZ* gene (Figure 8A). This construct was placed in the WT, ∆*ihfA,* P*tet*-driven *ihfA* at the *intS* locus, and P*tet*-driven *ihfA* G62E at the *intS* locus (containing the G-E substitution at the 62^nd^ position that abolishes DNA-binding ability of IHF). The results for each reporter are presented in Figures 8B–D. Overall, no significant change was observed, regardless of the genetic background. Since IHF is known to bind to either end of IS1 and to IS5C, these results strongly suggest that IHF exerts its effect via direct binding to IS1 and IS5 without altering the expression level of either IS element.

### 2.8 cAMP-Crp is a positive regulator of IS insertion when present upstream of P*bgl*

**Figure 9:**
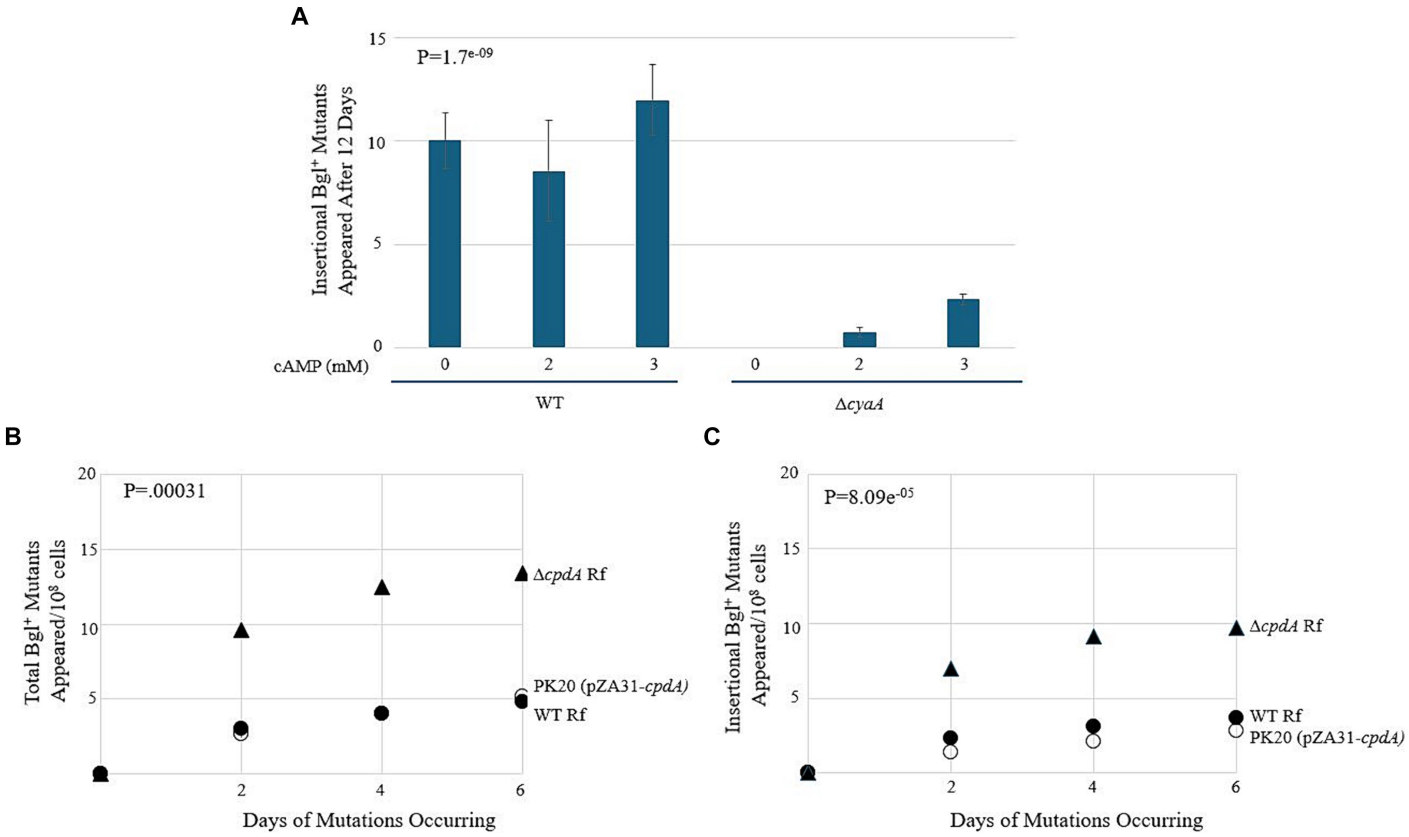
cAMP-Crp is a positive regulator of IS insertion into *bgl*. **(A)** Effect of adding external cAMP on insertional Bgl^+^ mutations in wild type cells and ∆*cyaA* cells. cAMP was added to M9 + Salicin agar from 0 mM to 3 mM. **(B,C)** Effects of *cpdA* deletion and overexpression on total Bgl^+^ mutations **(B)** and insertional Bgl^+^ mutations **(C)**. *cpdA* encodes a phosphodiesterase which degrades cAMP to 5’-AMP, and in its absence, cAMP levels increase. For overexpression, the strong constitutive *tet* promoter, P*tet*, was used to drive *cpdA* on the plasmid pZA31. WT Rf = ●; ∆*cpdA* Rf = ▲; PK20 = ○.

To test whether Crp (when activated by cAMP) affects the rate of insertional mutations in the upstream region, two types of mutation assays were conducted. Simply testing a ∆*crp* mutant is not an option in this case, as Crp is required for expression of the *bgl* operon. If insertion takes place in a *crp* deletion mutant, we would be unable to observe it since the colony would not grow. Therefore, we first decided to perform mutation assays using WT on M9 salicin plates with minimal cAMP and compare mutation rates to those growing on plates with an excess of cAMP. The results of this experiment are shown in Figure 9A. They demonstrate no change in insertional mutants as the amount of extracellular cAMP increases.

Next, we compared these WT results to those of a *cyaA* deletion mutant. *cyaA* encodes adenylate cyclase, and without it, the cells are unable to produce cAMP and must rely on extracellular cAMP provided in the growth media. In Figure 9A, we observe an increase in insertional mutants as the level of extracellular cAMP increases. Interestingly, the number of total colonies was like those observed for the WT strain, suggesting that the lack of *cyaA* has a significant effect on IS insertion specifically. The next set of experiments used *cpdA* to control the levels of intracellular cAMP. *cpdA* encodes a phosphodiesterase which degrades cAMP to 5’-AMP, and in its absence, cAMP levels increase. Similarly, if *cpdA* is expressed at higher levels, the amount of cAMP, and therefore the amount of active Crp, should decrease. A *cpdA* deletion mutant was constructed and was given an “empty” pZA31 plasmid for isogeneity, producing ∆*cpdA* Rf. A pZA31 plasmid containing P*tet-cpdA* was electroporated into WT cells, yielding PK20. All three strains were subjected to the *bgl* mutation assay as previously described. The results are presented in Figures 9B,C. The ∆*cpdA* mutant showed a more than two-fold increase in insertional mutants compared to the WT strain, and the *cpdA* overexpression strain PK20 showed a similar number of total colonies as WT, but with only half as many insertional mutants. Together, these data suggest that Crp, when activated by cAMP, is a positive regulator of insertion upstream of the *bgl* operon. In the absence of cAMP via deletion of *cyaA* or via increased degradation of cAMP, the insertion rate decreased 2–3-fold.

### 2.9 *ihfA* deletion or *ihfA*/*B* constitutive expression with differential cAMP-Crp levels

**Figure 10:**
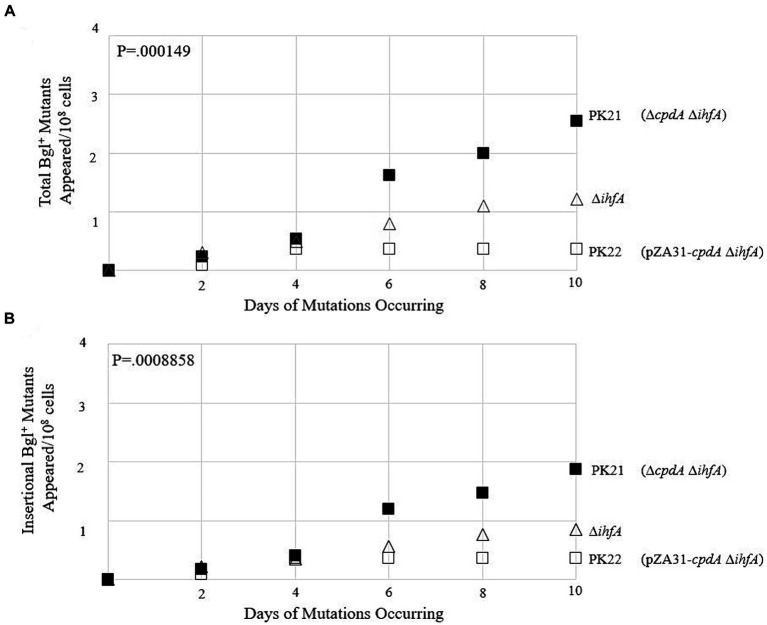
Effects of varying levels of cAMP on Bgl^+^ mutations in ∆*ihfA* cells. **(A)** Effects of *cpdA* deletion and overexpression on total Bgl^+^ mutations in ∆*ihfA* cells. **(B)** Effects of *cpdA* deletion and overexpression on insertional Bgl^+^ mutations in ∆*ihfA* cells. To elevate cellular cAMP levels, *cpdA* was deleted. To decrease cellular cAMP levels, *cpdA* was driven by P*tet* on plasmid pZA31.

To observe how loss of *ihfA* affects mutation frequencies along with differing cAMP-Crp complex levels, the ∆*cpdA* Rf and PK20 backgrounds were transferred to the ∆*ihfA* strain. The results of this set of mutation essays are shown in Figures 10A,B. In the absence of IHF, increased Crp-cAMP by deleting *cpdA* led to a more than 2-fold elevation of Bgl^+^ mutations. Similarly, decreased Crp-cAMP by overexpressing *cpdA* led to a 3-fold reduction of Bgl^+^ mutations in the absence of IHF. Combining both IHF and Crp, these two proteins affect Bgl^+^ mutations by up to 33-fold (Figures 9B, 10A).

## 3 Discussion

### 3.1 The effect of PK01, causing a decrease in the IS insertion rate

PK01, which replaced the native promoters of *ihfA* and *ihfB* with the constitutive P*tet* promoter, had the surprising effect of lowering the insertion rate into *bgl* by nearly two-fold compared to WT. Additionally, the titratable strain PK02_R, which grows similarly to WT, grew only around half as many colonies when saturated with its inducer aTC. We here provide a few possible explanations for these phenomena. The first and most likely possibility is that the native promoters for *ihfA* and *ihfB* are already strong. Since IHF is a global protein with several functions in the cell and with genes in locations adjacent to other important genes, promoters for both subunits may be very strong (Pozdeev et al., 2022). It may be that the promoters driving these subunits are already operating at a higher rate of expression than P*tet* itself. Therefore, in an effort to measure the mutational response of a cell by increasing expression, it is possible that replacement by P*tet*, while strong, could actually have expressed *ihfA* and *ihfB* at a lower rate than the native promoter. Our data on the titratable strains PK01_R and PK02_R are in support of this hypothesis, because even at maximal levels of inductions, the mutation rate remains lower than WT.

Another possibility is that this strain is in fact expressing IHF at levels higher than WT, but that this has an overall negative effect on mutation frequency. For example, these increased levels of expression may be toxic to the cell, or the increased binding of IHF to the multitude of its recognized binding sites may have pleiotropic effects that lead to lower growth rates, and consequently less insertion.

A final possibility is related to the fact that IHF levels increase greatly when the cell is starving (Bushman et al., 1985; Ditto et al., 1994). It may be that this increase is regulated by transcription factors affecting the native *ihfA*/*ihfB* promoters under starvation conditions such as during the mutation assay. If we change the promoters to P*tet*, we may well have inadvertently removed the cell’s normal ability to respond to starvation by upregulating *ihfA* and *ihfB* expression, which would also bring IHF levels to a lower-than-expected value.

### 3.2 Effect of *cyaA* deletion on the IS1/5 insertion rate

cAMP-activated Crp, which is known to bind in *bgl*’s promoter region and positively regulate transcription, was also shown to positively regulate the rate of IS element transposition upstream of *bgl*. Results of a mutation assay comparing WT with an adenylate cyclase deletion mutant (∆*cyaA*) showed a decrease in the incidence of insertional colonies, although the overall Bgl^+^ colony counts remained comparable. Similarly, in backgrounds overexpressing or deleting the cAMP-degrading phosphodiesterase gene, *cpdA*, the rate of insertion decreased or increased, respectively. Thus, these results reveal a new role for Crp within the context of *bgl* as a regulator of transposition as well as transcription. Interestingly, previous data from our group have suggested that in the *glpFK* operon, cAMP-Crp has a negative effect on upstream IS insertion. It appears that while Crp maintains its status as a positive transcriptional regulator in the vast majority of operons which it influences, its role with respect to mutational changes in the *bgl* operon may be responsive to the sequences and/or DNA structures surrounding its binding site.

Crp has a known binding site in *bgl*’s promoter region, and this site is close to or overlapping the projected SIDD site into which IS elements insert (Humayun et al., 2017). This novel effect of cAMP-activated Crp presented here likely results from the increased energetic favorability for insertion to take place due to its binding. However, advances in computational programs that analyze energetics would be helpful to confirm this, as the current programs do not account for the binding of proteins to the DNA.

### 3.3 Strength of the IS1 promoter region

The IS1-*lacZ* reporter activity in section 2.7 proved to be low, even when compared to the two orientations of the IS5 reporter. After a thorough examination of the sequence of the construct, no mutation was found, leading us to consider other possibilities as to why IS1 exerted such a low operon transcriptional activity compared to IS5. We submit that either the growth conditions were not sufficient to increase IS1’s promoter to higher levels, or the region upstream of the IS1-*lacZ* gene construct lacks a site for a postulated positive transcription factor for the IS1 promoter. If so, it must be present in the other native locations in the genome where IS1 exists. Both considerations may independently or together explain why IS1’s reporter showed such low activity.

### 3.4 Effect of IHF via DNA conformation of the IS element to allow for efficient transposase binding

As stated in the Results section, both ends of IS1 and at least one end of IS5 contain IHF binding sites (Gamas et al., 1987; Prentki et al., 1987; Muramatsu et al., 1988). However, LacZ assays measuring the activities of the IS1 and IS5 promoters showed no clear change when the level of IHF binding was altered. Despite this, our other data indicate a non-specific effect of IHF on transposition of IS1 and IS5. Thus, the effect exerted by IHF on these IS elements must be of a nature other than transcriptional. Both IS1 and IS5 have DDE catalytic regions (Ohta et al., 2002; Curcio and Derbyshire, 2003), and may therefore act similarly for the purposes of replicative excision from the *E. coli* genome. Our IHF mutation assay data supports this; if IHF only affected one element’s transposition and not the other, then only a 2-fold change at best would be expected, and all the insertional colonies would contain the same element, instead of the roughly equal levels of IS1 and IS5 insertion that has been classically observed (Schnetz and Rak, 1988; P.K., unpublished data). Work on other transposons with DDE activity has suggested that IHF plays an important, but not essential, role in the efficient transposition of Tn10 and the Mu bacteriophage (Kobryn et al., 2002; Swinger and Rice, 2004; Gueguen et al., 2005). The role of IHF involves assisting in the conformation of the transposon via binding so that the transposase protein can effectively bind to the transposon ends and then dimerize (or tetramerize in the case of Mu). It has been shown that when IHF is not present, DNA supercoiling has a compensatory effect, allowing for some transposition to occur even when an important conformational factor is not present (Chalmers et al., 1998). Interestingly, Tn10 *in vitro* experiments mimicking *in vivo* supercoiling levels in the absence of IHF observed a ~ 10-fold decrease in transposition frequency (called the “basal level” of 27 excision), which is a similar decrease to what we observed in the *ihfA* deletion mutant (∆*ihfA*) for the Bgl^+^ mutation assay (Chalmers et al., 1998; Figure 2A). We utilized *in vivo* models to support past findings in relation to other transposable elements and draw further similarities between IS elements and other transposons in the DDE transposase family.

## 4 Conclusion

In this work, we demonstrate that IHF and Crp are positive regulators of IS1 and IS5 insertion. Without both functional IHF subunits and the ability to bind DNA, a strong decrease in total Bgl^+^ mutations is observed, and an even larger decrease in insertional Bgl^+^ mutations. No matter the amount of antiterminator protein BglG, this strong decrease is still observed. This effect extends to other operons where IS1 and IS5 insertion takes place. We show that the effect of IHF is not transcriptional in nature - that is, binding of IHF to the IS element causes no significant change in transposase gene expression. Therefore, we suggest that IHF’s role may instead be related to assisting DNA conformation or transpososome formation at the site of the IS element. We show that increasing the amount of extracellular cAMP increases insertional Bgl^+^ mutations. Additionally, by increasing intracellular cAMP levels via *cpdA* deletion, a more than 2-fold increase in insertional Bgl^+^ mutations is observed. IHF and Crp’s effects are independent of each other, and combining their effects causes an up to 33-fold change in insertional mutations.

## 5 Materials and methods

### 5.1 Construction of strains used

#### 5.1.1 Construction of deletion mutants

CGSC strains JW3964-1, JW0430-3, JW1702-1, and JW3000-1 (*E. coli* Genetic Stock Center, Yale Univ.) carry the deletion mutations of *hupA*, *hupB*, *ihfA*, and *cpdA*, respectively. For each of these mutants, a kanamycin resistance (*km^r^*) gene was substituted for the target gene. These mutations were individually transferred to strain BW25113 (wild type/WT) (Datsenko and Wanner, 2000) by P1 transduction, and the *km^r^* gene was subsequently flipped out by pCP20 (Datsenko and Wanner, 2000), yielding deletion mutant strains Δ*hupA*, Δ*hupB*, Δ*ihfA*, and Δ*cpdA*, respectively (Supplementary Table S1). Using P1 transduction, the *ihfA* mutation was transferred into the *crp* deletion strain Δ*crp* (Zhang and Saier, 2009b), yielding the Δ*crp*Δ*ihfA* double mutant. The same *ihfA* mutation was transferred into strain Δ*cpdA*, yielding the Δ*cpdA*Δ*ihfA* double mutant.

#### 5.1.2 Construction of *cpdA* overexpression plasmid

The *cpdA* gene was amplified from BW25113 chromosomal DNA using oligos cpdA-Kpn-F and cpdA-Bam-R (Supplementary Table S2). The PCR products were gel purified, digested with KpnI/BamHI and then ligated into the same sites of pZA31P*tet* (Lutz and Bujard, 1997) yielding pZA31-*cpdA*. pZA31-*cpdA* was transformed into strains BW23113 and Δ*ihfA*, yielding PK20 and PK22, respectively (Supplementary Table S1). Plasmid pZA31 P*tet*-Rf (Levine et al., 2007) carries a random fragment (RF) and was used as a negative control (WT Rf).

#### 5.1.3 Construction of P*tet* driving *ihfA*, *ihfB*, and *ihfA*G62E on the chromosome

Using plasmid pKDT:P*tet* (Klumpp et al., 2009) as a template, the cassette “*km^r^*:*rrnB*T:P*tet*,” containing the *km^r^* gene, the *rrnB* terminator (*rrnB*T) and the P*tet* promoter, was amplified using the primer pair Ptet-ihfA-P1 and Ptet-ihfA-P2 (Supplementary Table S2). Using lambda-red system (Datsenko and Wanner, 2000), the PCR products were integrated into the chromosome of BW25113 to replace the “CCT” nucleotides immediately upstream of the *ihfA* translational start point. Chromosomal integration was confirmed, first by colony PCR, and subsequently by DNA sequencing. This yielded strain P*tet*-*ihfA*, in which the strong *tet* promoter drives expression of the *ihfA* gene. Similarly, the “*km^r^*:*rrnB*T:P*tet*” cassette amplified by Ptet-ihfB-P1 and Ptet-ihfB-P2 (Supplementary Table S2) from pKDT:P*tet* was substituted for the *ihfB* upstream promoter region (−46 to −1 relative to the *ihfB* translational start point), yielding strain P*tet*-*ihfB*.

To make an *ihfA*/*ihfB* double overexpression strain, the *km^r^* gene was first flipped out from strain P*tet-ihfB* by pCP20. The cassette of “*km^r^*:*rrnB*T:P*tet*” driving *ihfA* gene from strain P*tet*-*ihfA* was then transferred to Km-sensitive P*tet*-*ihfB* by P1- transduction, yielding strain PK01 (Supplementary Table S1), in which both *ihfA* and *ihfB* are simultaneously driven by P*tet*. To titrate expression of *ihfA* and *ihfB*, the transcription unit including a constitutively expressed *tetR* gene and a spectinomycin resistance (*sp^r^*) marker was transferred to PK03 by P1-transduction as mentioned above, yielding strain PK01_R.

To further increase *ihfA* expression, a second copy of P*tet* driving *ihfA* was inserted to another chromosomal location. To do so, the *km^r^*:*rrnB*T:P*tet*-*ihfA* expression cassette was amplified from the genomic DNA of strain P*tet*-*ihfA* using primers intS1-P1 and ihfA2-P2. The products were integrated into the *intS* site to replace the region between −229 and + 1,101 (relative to the *intS* translational initiation site). The chromosomal integration was confirmed by colony PCR and subsequently by DNA sequencing, yielding strain P*tet*-*ihfA* @*intS*.

The Glycine residue at position 62 is required for IhfA to bind to the DNA (Granston and Nash, 1993; Hales et al., 1994). To reduce or abolish IhfA’s DNA-binding ability, this residue was changed to a glutamate residue using fusion PCR. The first part (5′ region) of *ihfA* was amplified from BW25113 gnomic DNA using ihfA-Kpn-F and ihfA.G62E-R (carrying the G62E alteration). The second part (3′ region) was amplified using ihfA.G62E-F (carrying the G62E alteration and overlapping ihfA.G62E-R) and ihfA-Bam-R. Both products were gel purified and fused together using primers ihfA-Kpn-F and ihfA-Bam-R. The fused products (that is, ihfA.G62E) were gel purified, digested with KpnI and BamHI, and then ligated into the same sites of pKDT_P*tet*, yielding pKDT_P*tet*-*ihfA*.G62E. The cassette “*km^r^*:*rrnB*T:P*tet*-*ihfA*.G62E” was amplified using intS-P1 and ihfA-P2, gel purified and then integrated into the *intS* site as for P*tet*-*ihfA* @*intS*. The chromosomal integration was confirmed by colony PCR and subsequently by DNA sequencing, yielding strain PK10, which still maintains the native *ihfA* but constitutively expresses the modified *ihfA*.G62E at the *intS* locus. This cassette was transferred to an *ihfA* deletion background by P1 transduction, yielding PK11.

To over express *ihfA* and *ihfB* simultaneously, both structural genes were first PCR amplified individually from *E. coli* genomic DNA. After being gel purified, these two DNA fragments were fused together by PCR. There is a 20 bp intergenic region with nucleotide sequences “tctgattAGAGGAaacagct” between these two genes. The capitalized nucleotides refer to the RBS site for *ihfB*, which is the same as one for *ihfA* located within P*tet*. The fusion *ihfA*/*ihfB* products were digested with KpnI and BamHI and then ligated into pZA31P*tet* digested with the same enzymes, yielding pZA31-*ihfAB*, in which *ihfA* and *ihfB* are driven by P*tet* and both genes have the same RBS site.

### 5.2 Construction of the IS1 and IS5 promoter *lacZ* reporters

The promoter region (−55 to +30 relative to the *insA* translation initiation site) driving the IS1 transposase gene *insAB1* was amplified using IS1p-Xho-F and IS1pBam-R from BW25113 genomic DNA. This region contains all of the upstream region (including the left-hand IR) and the first 10 residues of *insA* (plus a stop codon TAA). The amplified products were digested with XhoI and BamHI and cloned into the same sites of the default integration vector, pKDT (Klumpp et al., 2009), yielding pKDT-PIS1. The region carrying the *km^r^*, *rrnB*T and PIS1 (*km^r^*:*rrnB*T:PIS1) was PCR amplified using oligos IS1- Z-P1 and IS1-Z-P2 (Supplementary Table S2) and then integrated into the chromosomal default strain EQ42 (Klumpp et al., 2009) to replace the *lacI* gene and the *lacZ* promoter. The resultant strain carries the *km^r^*:*rrnB*T:PIS1 cassette followed by *lacZ*’s ribosomal binding site (RBS) and the *lacZ* structural gene within the *lac* locus. After being confirmed by PCR and sequencing, the promoter reporter, PIS1, driving *lacZ* expression (that is, PIS1-*lacZ*) was transferred into BW25113 and various genetic backgrounds by P1 transduction. This yielded the IS1 promoter reporter strains IS1-Z, *ΔihfA*_IS1-Z, PK14, and PK15, respectively.

IS5 carries three open reading frames (*ins5A*, *ins5B*, and *ins5C*). *ins5A* encodes the main transposase, and it is transcribed from its own promoter located close to the left-hand IR while the divergent *ins5B* and *ins5C* genes may form an operon that is driven by another promoter located close to the right-hand IR (Sawers, 2005). The *ins5A* promoter region (−68 to +30 relative to the *ins5A* translational initiation site) and the *ins5CB* promoter region (−207 to +30 relative to the *ins5C* translational initiation site) were individually cloned into pKDT, yielding pKDT_PIS5A and pKDT_PIS5C, respectively. As for PIS1 described above, PIS5A and PIS5CB each carries the first 10 amino acids from the target gene followed by a stop codon TAA. These promoters were integrated into the *lac* site on BW25113 chromosome as for PIS1-Z, yielding strains IS5A-Z and IS5C-Z, respectively. Using P1 transductions, these new IS5 promoter *lacZ* reporters were transferred to other genetic backgrounds by P1 transduction. This yielded the IS5 transposase promoter (PIS5A) reporter strains *ΔihfA*_IS5A-Z, PK16, and PK17, as well as the IS5C promoter reporter strains Δ*ihfA*_IS5C-Z, PK18, and PK19.

### 5.3 β-galactosidase (LacZ) assays

*Escherichia coli* reporter strains were cultured in 4 mL of LB contained in glass test tubes (1.5 cm in diameter × 15 cm in length) with shaking at 37°C for 8 h. An amount of 30 μL of LB cultures were used to inoculate 3 mL of M63 minimal media in smaller glass tubes (1.2 cm × 12 cm), and the tubes were shaken at 37°C overnight. The carbon sources were 0.5% glycerol, 0.5% salicin, or both. The tubes were rotated at 250 rpm and 37°C, and cell densities (OD_600_) were measured with a Bio-Rad spectrophotometer. During the exponential growth phase, four samples were collected in the range of OD_600_ from 0.1 to 1. The samples (roughly 0.3 mL for promoter reporter strains, 0.9 mL for the IS1/5 promoters), and 0.6 mL for operon reporter strains were immediately frozen at −20°C prior to β-galactosidase assays. To measure β-galactosidase activities in *bgl* promoter reporter strains, 0.8 mL of Z-buffer containing β-mercaptoethanol (2.7 μL/mL) and sodium dodecyl sulfate (SDS) (0.005%) was mixed with 0.2 mL of sample and 25 μL of CHCl_3_ in test tubes. Alternatively, for *bgl* operon reporter strains, 0.5 mL of Z-buffer was mixed with 0.5 mL of the sample. The tubes were vortexed twice (each time for 10 s at a constant speed) and incubated in a 37°C water bath until temperature equilibration. A 0.2 mL aliquot of O-nitrophenyl galactoside (ONPG) substrate (4 mg/mL) was then added to each test tube. When a yellow color developed, the reaction was stopped by adding 0.5 mL of 1 M Na_2_CO_3_ followed by vortexing. Reaction mixtures were centrifuged (15,000 rpm, 3 min), and the absorbance values of the supernatants were measured at 420 nm and 550 nm. A control tube was run in parallel using M63 salts instead of the test sample. β-galactosidase activity (Miller units) = [(OD_420_–1.75 × OD_550_)/(sample volume in mL × time in min)] × 1,000 (Miller, 1972). For a given test strain, the slope of OD_600_ values versus β-galactosidase activities was referred to as the promoter activity or the operon activity.

To test the effect of IHF on *bglGFB* transcription, the *bgl* promoter reporter (P*bgl*-Z) and the *bgl* operon reporter (P*bgl*-G-Z) (Tran et al., 2022) were individually transferred to an *ihfA* deletion background. This yielded strains ∆*ihfA*_P*bgl*-Z and ∆*ihfA*_P*bgl*-G-Z.

### 5.4 β-glucosidase (BglB) assay

β-glucosidase assay was performed as described in our previous study (Lam et al., 2022). Briefly, 20 μL of LB culture was transferred to M63 minimal medium with 0.66% casamino acids (CAA). After overnight growth at 37°C with shaking, the culture was diluted into 6 mL of M63 + 0.66% CAA +0.5% salicin minimal medium at a starting OD_600_ of 0.025. The cells were grown at 37°C with shaking. Five samples with 0.8 mL each were collected during the late exponential growth phase when the *bgl* operon was fully induced with an OD_600_ of above 1.5. The samples were centrifuged at a speed of 5,500 rpm for 2.5 min and the cells were suspended with 1 mL of Z-buffer with 50 μg/mL chloramphenicol.

To measure β-glucosidase (BglB) activities, test samples were warmed in a 37°C water bath, and 200 μL of *p*-nitrophenyl-β-D-glucoside (PNPG, 8 mg/mL) was added to the cell suspension in Z-buffer. After a visible yellow color appeared, the reaction was terminated by adding 0.5 mL of 1 M Na_2_CO_3_ and subsequently vortexing. The reaction mixture was centrifuged, and the absorbance of the reaction mixture was measured at 420 nm and 550 nm. The BglB activity of each sample was calculated using the equation: β-glucosidase (BglB) activity = [1,000 × (OD_420 nm_ – 1.75 × OD_550 nm_) × Dilution factor]/[OD_600 nm_ × Time of reaction (min) × Volume of sample (mL)]. The activity of the strain was determined by averaging the BglB activities of the samples measured.

β-glucosidase (BglB) assays were conducted in wild type and ∆*ihfA* backgrounds. To see the IHF effect on the cells with higher operon expression, strains G50 (deleted for the two terminators flanking *bglG*) and ∆P*bgl*-G (deleted for P*bgl* and *bglG* together with two terminators; Lam et al., 2022) was used.

### 5.5 Bgl^+^ mutation assays

Bgl^+^ mutation assays were performed on minimal M9 agar plates with 0.5% of a β-glucoside (salicin) as the sole carbon source. Strains to be tested (from single fresh colonies) were cultured in LB liquid medium for approximately 7 h at 37°C, washed twice with carbon source-free M9 salts (M9) and applied onto plates (2 × 10^7^ cells/plate). The plates were then incubated in a 30°C incubator and were examined every 2 days for the appearance of Bgl^+^ colonies, with each colony representing a new Bgl^+^ mutation. On these β-glucoside minimal agar plates, any colonies appearing by day 2 were considered to be from Bgl^+^ cells initially applied onto the plates. They were therefore subtracted from the subsequent measurements. IS1 and IS5 have been previously shown to insert into the same upstream *bgl* promoter region. Colony PCR using primers flanking this region was used to differentiate between insertional and non-insertional mutants. Mutation frequency was determined in the manner described in Cairns and Foster (1991) and Zhang and Saier (2009b).

For the mutation assays which used differential levels of aTC, the growth media was prepared with different amounts of aTC to obtain several ng/mL concentrations as outlined in Results 2.3.

To determine the effects of other carbon sources on Bgl^+^ mutations, mutation assays were performed on minimal M9 agar plates with 0.25% glycerol or 1% propanediol as the sole carbon source. To determine the total populations, the cells were washed off the minimal M9 agar plates at relevant time points, serially diluted, and plated onto LB agar plates. To determine Bgl^+^ populations, appropriate dilutions were applied on M9 + salicin agar plates. The frequencies of Bgl^+^ mutations were determined as described above for Bgl^+^ on M9 + salicin agar plates.

### 5.6 Glp^+^ mutation assays

Glp^**+**^ mutation assays were conducted on glycerol M9 minimal agar plates as described in Zhang and Saier (2009b). Strains ∆*crp* and ∆*crp*∆*ihfA* were used for the mutation assays.

### 5.7 Swarming mutation assays

Using the wild-type and Δ*ihfA* strains, the swarming mutation assays for the appearance of hyper-swarming mutants (outgrowing subpopulations from the inoculated cells) were carried out using the method of Barker et al. (2004). Briefly, overnight cell cultures in LB media were washed once with M9 salts and diluted to an OD_600_ of 1.0 prior to use. Two microliters of the cell suspensions were streaked across the centers of LB semisolid (0.3% agar) plates (diameter = 9 cm) using a plastic transfer loop. The plates were incubated at 30°C. The swarming mutants, represented by outgrowths of motile subpopulations from the streaked cells, were counted. The mutation frequency was normalized as outgrowths (mutations) per 9-cm cell streak. Insertional mutants were verified via colony PCR.

### 5.8 Propanediol growth mutation assay

The assay for PPD^**+**^ mutations was conducted by applying cell suspensions from fresh overnight cultures onto propanediol (1%) M9 minimal agar plates (~10^8^ cells/plate). The wild-type and other strains were tested over a period of several days as in the Bgl^+^ mutation assay. Total populations and mutation frequency were determined as described above under “Bgl^+^ mutation assays.”

### 5.9 Statistical analysis

The program R was used for all statistical analyses. *p*-values were generated to determine statistical significance, with values under 0.05 treated as statistically significant. The strains tested were plated along with known controls for each experimental run. Results shown are from a minimum of 3 separate runs/strain, with each run consisting of several plates. The data reported on the figures are expressed as the mean of a minimum of three runs for each strain at each time point. LacZ activity assay results are expressed as the slope of a line generated from 4 time points/run and a minimum of 2 runs (at least 8 data points total). BglB assay results are expressed as the mean of 12 data points from a minimum of 3 separate runs.

## Data availability statement

The original contributions presented in the study are included in the article/Supplementary material, further inquiries can be directed to the corresponding authors.

## Author contributions

PK: Data curation, Investigation, Methodology, Writing – original draft, Writing – review & editing. ZZ: Conceptualization, Data curation, Methodology, Supervision, Writing – original draft, Writing – review & editing. MS: Conceptualization, Funding acquisition, Project administration, Supervision, Writing – review & editing.
