# Supplementary material for: The effect of DNA-binding proteins on insertion sequence element transposition upstream of the bgl operon in Escherichia coli
Source: Front Microbiol. 2024 Apr 11;15:1388522. doi: 10.3389/fmicb.2024.1388522 (PMC11043490; doi:10.3389/fmicb.2024.1388522)
Supplement: Supplementary file 1 [file Data_Sheet_1.docx]

**Supplementary Information**

**
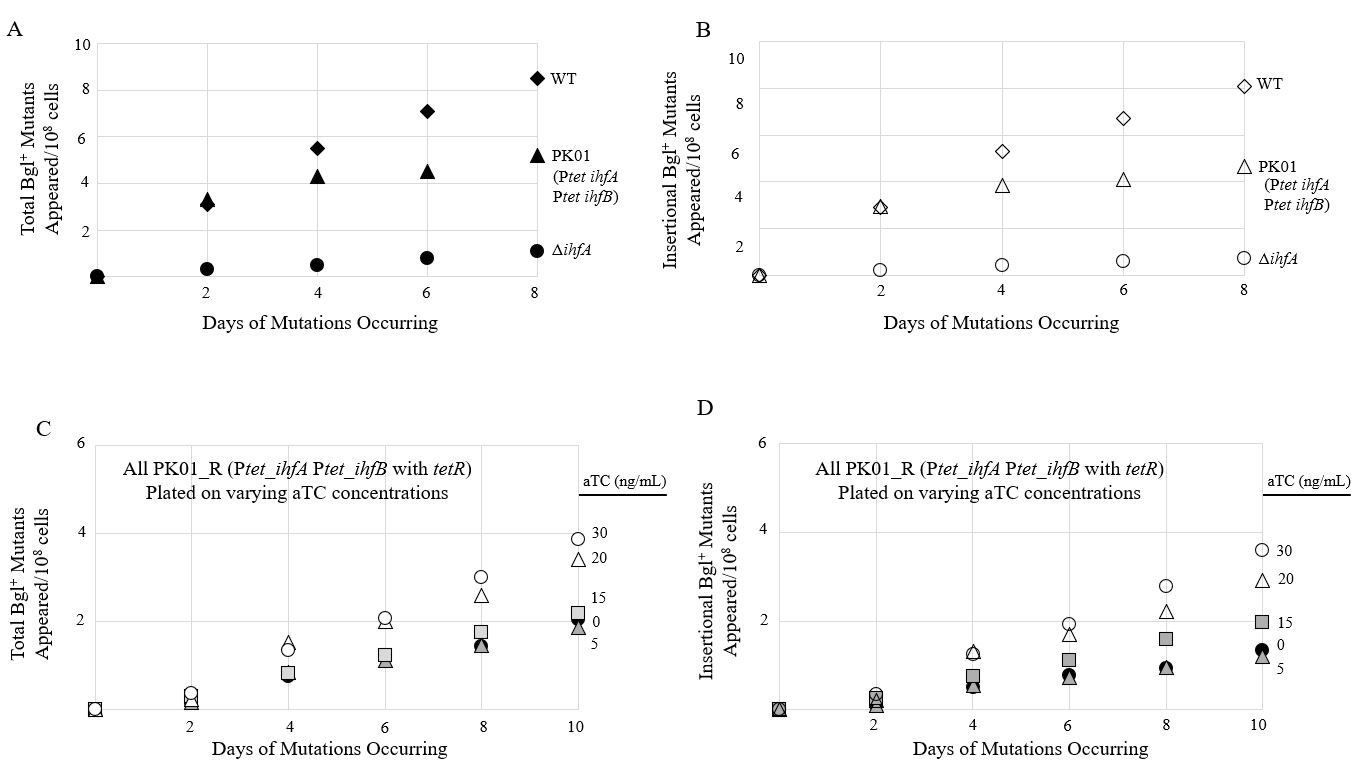
**

**Figure S1- P*tet*-driven expression of *ihfA* and *ihfB* in the genome slightly lowers the IS insertional rate into *bgl*.**

**A) Effect of P*tet*-driven chromosomal *ihfA* and *ihfB* expression on total Bgl^+^ mutations. B) Effect of P*tet*-driven chromosomal *ihfA* and *ihfB* expression on insertional Bgl^+^ mutations. C) Effect of titrating chromosomal *ihfA* and *ihfB* expressions on total Bgl^+^ mutations. D) Effect of titrating chromosomal *ihfA* and *ihfB* expressions on insertional Bgl^+^ mutations. For Figures C and D, mutation assays were performed as previously described on M9 salicin agar plates containing aTC at 0 to 30 ng/ml over a 12-day period. 0aTC= ●; 5aTC= ▲; 15aTC= ■; 20aTC= ∆; 30aTC= ○.**

**
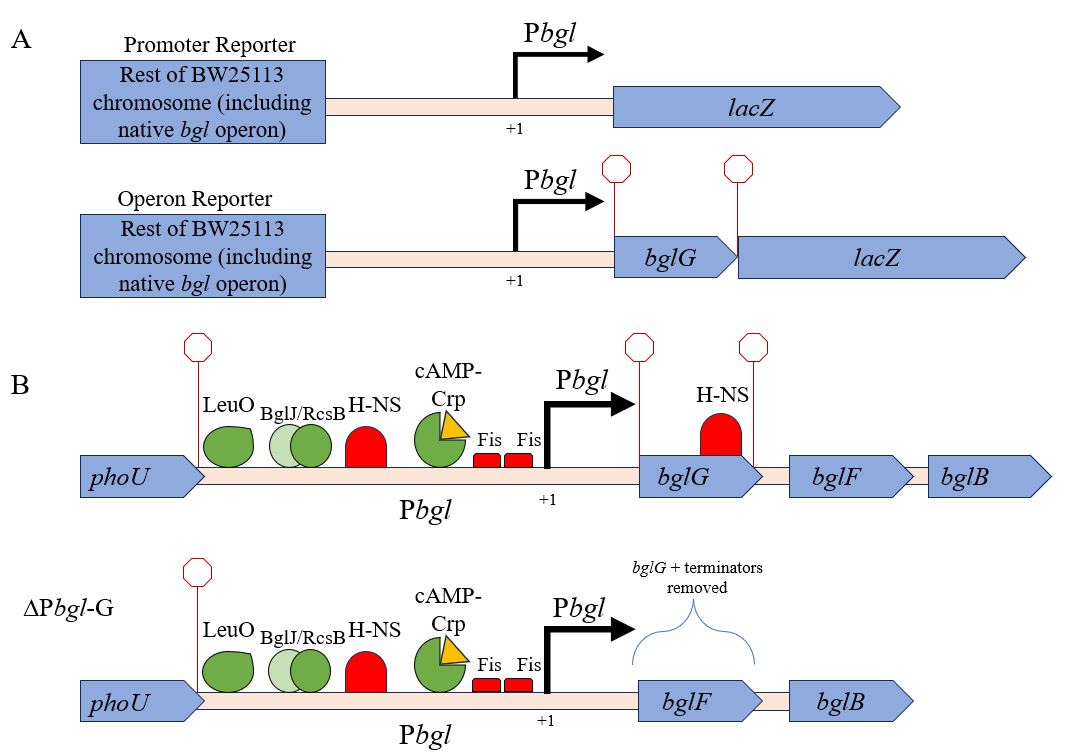
**

**Figure S2- Construction of strains used to measure *bgl* transcriptional activity.**

**A) Portions of a separate *bgl* operon were placed in front of *lacZ* at the *lac* locus, leaving the native *bgl* operon unchanged to allow normal induction by the substrate. This secondary *bgl* reporter contained either the *bgl* promoter region alone to measure promoter activity, or the promoter region followed by *bglG* to measure operon activity. B) The native *bgl* operon was measured for Figure 5C. Strain ∆P*bgl*-G was constructed to increase overall transcriptional activity and see any potential changes more clearly in Figure 5D. In the ∆P*bgl*-G strain, the *bglG* gene and both terminators are deleted downstream of the *bgl* promoter, but the rest of the operon remains as before.**
