# Supplementary material for: The effect of DNA-binding proteins on insertion sequence element transposition upstream of the bgl operon in Escherichia coli
Source: Front Microbiol. 2024 Apr 11;15:1388522. doi: 10.3389/fmicb.2024.1388522 (PMC11043490; doi:10.3389/fmicb.2024.1388522)
Supplement: Supplementary file 2 [file Table_1.docx]

1. **Supplementary Table S1. Strains and plasmids used in this study.**

| **Strain or plasmid** | **Genotype or description** | **Reference** |
| --- | --- | --- |
| **Strains** |  |  |
| BW25113 | *E. coli* K12 strain, wild type (WT) | Datsenko & Wanner, 2000 |
| Δ*stpA* | Δ*stpA* in BW25113 | Tran et al., 2022 |
| Δ*hupA* | Δ*hupA* in BW25113 | This study |
| Δ*hupB* | Δ*hupB* in BW25113 | This study |
| Δ*ihfA* | Δ*ihfA* in BW25113 | This study |
| Δ*bglG* | Δ*bglG* in BW25113 | Tran et al., 2022 |
| Δ*bglJ* | Δ*bglJ* in BW25113 | Tran et al., 2022 |
| Δ*leuO* | Δ*leuO* in BW25113 | Tran et al., 2022 |
| Δ*rcsB* | Δ*rcsB* in BW25113 | Tran et al., 2022 |
| Δ*fis* | Δ*fis* in BW25113 | Tran et al., 2022 |
| Δ*ihfB* | Δ*ihfB* in BW25113 | This study |
| P*tet*-G | P*tet*-driven *bglG* at the *intS* locus; the native *bgl* operon is intact | Zhang et al., 2022 |
| P*tet*-G_Δ*ihfA* | Δ*ihfA* in Ptet-G | This study |
| Iq-G | *lacIq*-driven *bglG* at the *intS* locus; the native *bgl* operon is intact | Tran et al., 2022 |
| Iq-G_Δ*ihfA* | Δ*ihfA* in Iq-G | This study |
| PK01 | P*tet*-driven *ihfA* and P*tet-*driven *ihfB* at native chromosomal loci | This study |
| PK01_R | Constitutive expression of *tetR* in PK01 | This study |
| PK02 | P*tet*-driven *ihfA* and *ihfB* together on pZA31 | This study |
| PK02_R | Constitutive expression of *tetR* in PK04 | This study |
| WT Rf | BW25113 with an empty pZA31 plasmid (control) | This study |
| P*bgl*-Z | P*bgl* driven *lacZ* at the *lac* locus. The native *bgl* operon is intact | Tran et al., 2022 |
| P*bgl*-G-Z | P*bgl* driven *bglG* and *lacZ* at the *lac* locus. The *bgl* operon is intact | Tran et al., 2022 |
| ∆*ihfA*_P*bgl*-Z | ∆*ihfA* in P*bgl*-Z | This work |
| ∆*ihfA*_P*bgl*-G-Z | ∆*ihfA* in P*bgl*-G-Z | This work |
| ∆*bglGFB* | ∆*bglG*, ∆*bglF* and ∆*bglB* in BW25113 | Lam et al., 2022 |
| IS5Bgl^+^ | An IS5 insertional Bgl^+^ mutant | This work |
| PK03 | ∆*ihfA* in an IS5 insertional Bgl^+^ mutant | This work |
| G50 | Truncated *bglG* and deletion of two terminators flanking *bglG* | Lam et al., 2022 |
| PK04 | ∆*ihfA* in G50 | This work |
| PK05 | P*tet*-driven *ihfA* and *ihfB* gene at the *intS* locus in G50 | This work |
| ∆P*bgl*-G | Deletion of P*bgl*, *bglG* and two terminators flanking *bglG* | Lam et al., 2022 |
| PK06 | ∆*ihfA* in ∆P*bgl*-G | This work |
| PK07 | ∆*fis* in ∆P*bgl*-G | This work |
| PK08 | ∆*ihfA* and ∆*fis* in ∆P*bgl*-G | This work |
| PK09 | P*tet*-driven *ihfA* and *ihfB* at the *intS* locus in ∆P*bgl*-G | This work |
| ∆*crp* | ∆*crp* in BW25113 | Zhang & Saier, 2009b |
| ∆*crp*∆*ihfA* | ∆*ihfA* and ∆*crp* in BW25113 | This study |
| PK10 | P*tet* driven *ihfA* containing the G62E mutation at the *ints* locus | This study |
| PK11 | ∆*ihfA* in PK10 | This study |
| PK12 | ∆*crp* in PK10 | This study |
| PK13 | Δ*crp* and ∆*ihfA* in PK10 | This study |
| IS1-Z | The IS1 transposase promoter driven *lacZ* at the *lac* locus | This work |
| ∆*ihfA*_IS1-Z | ∆*ihfA* in IS1-Z | This work |
| PK14 | P*tet*-driven *ihfA* gene at the *intS* locus in IS1-Z | This work |
| PK15 | P*tet*-driven *ihfA*_G62E at the *intS* locus in IS1-Z | This work |
| IS5A-Z | The IS5 transposase promoter driven *lacZ* at the *lac* locus | This work |
| ∆*ihfA*_IS5A-Z | ∆*ihfA* in IS5A-Z | This work |
| PK16 | P*tet*-driven *ihfA* gene at the *intS* locus in IS5A-Z | This work |
| PK17 | P*tet*-driven *ihfA*_G62E at the *intS* locus in IS5A-Z | This work |
| IS5C-Z | The *ins5BC* promoter driven *lacZ* at the *lac* locus | This work |
| ∆*ihfA*_IS5C-Z | ∆*ihfA* in IS5C-Z | This work |
| PK18 | P*tet*-driven *ihfA* gene at the *intS* locus in IS5C-Z | This work |
| PK19 | P*tet*-driven *ihfA*_G62E at the *intS* locus in IS5C-Z | This work |
| Δ*cyaA* | Δ*cyaA* in BW25113 | Zhang & Saier, 2016 |
| ∆*cpdA* Rf | ∆*cpdA* in BW25113 with an empty pZA31 plasmid | This work |
| PK20 | BW25113 with pZA31 containing P*tet*-driven *cpdA* | This work |
| PK21 | ∆*cpdA* Rf in ∆*ihfA* | This work |
| PK22 | PK20 in ∆*ihfA* | This work |
| **Plasmids** |  |  |
| pZA31 | Cloning vector with Ptet, *p15A* ori, Cm^r^ | Lutz and Bujard,1997 |
| pKDT | A *rrnB* terminator (*rrnB*T) in pKD13 | Klumpp et al., 2009 |
| pZA31-*ihfAB* | P*tet* driven *ihfA* and *ihfB* on pZA31 | This study |
| pZA31-*cpdA* | P*tet* driven *cpdA* on pZA31 | This study |
| pKDT_Ptet-*ihfA*.G62E | pKDT carrying Ptet driven *ihfA*.G62E | This study |
| pKDT_PIS1 | pKDT carrying the IS1 transposase promoter | This study |
| pKDT_PIS5A | pKDT carrying the IS5 transposase promoter | This study |
| pKDT_PIS5C | pKDT carrying the *ins5BC* promoter | This study |

**References**

Datsenko, K. A., & Wanner, B. L. (2000). One-step inactivation of chromosomal genes in Escherichia coli K-12 using PCR products. *Proceedings of the National Academy of Sciences of the United States of America*, *97*(12), 6640–6645.

Zhang, Z., and Saier, M. H. (2016). Transposon-mediated activation of the *Escherichia coli* *glpFK* operon is inhibited by specific DNA-binding proteins: implications for stressinduced transposition events. Mutat. Res. 793-794, 22–31.

Lutz, R., & Bujard, H. (1997). Independent and tight regulation of transcriptional units in Escherichia coli via the LacR/O, the TetR/O and AraC/I1-I2 regulatory elements. *Nucleic acids research*, *25*(6), 1203–1210.

Klumpp, S., Zhang, Z., & Hwa, T. (2009). Growth rate-dependent global effects on gene expression in bacteria. *Cell*, *139*(7), 1366–1375.
