# Supplementary material for: The effect of DNA-binding proteins on insertion sequence element transposition upstream of the bgl operon in Escherichia coli
Source: Front Microbiol. 2024 Apr 11;15:1388522. doi: 10.3389/fmicb.2024.1388522 (PMC11043490; doi:10.3389/fmicb.2024.1388522)
Supplement: Supplementary file 3 [file Table_2.docx]

**Supplementary Table S2. Oligonucleotides used in this study.**

| Name | Sequence | Use |
| --- | --- | --- |
| Pbgl-F2 | tggcgatgagctggataaactgctg | Verification of IS1/IS5 insertion into Pbgl |
| Pbgl-R2 | tcagttcatgactgctcaaggcatac | Verification of IS1/IS5 insertion into Pbgl |
| 1. cpdA-Kpn-F | ataggtaccatggaaagcctgttaacccttcctctggctg | Cloning *cpdA* into pZA31P*tet* |
| 1. cpdA-Bam-R | aatggatcctcagtagccttctgaagcggtatcaggttg | Cloning *cpdA* into pZA31P*tet* |
| 1. cpdA-ver-F | aagcgcgtgtttattggtgagcaatg | Verification of *cpdA* cloning |
| 1. cpdA-ver-R | actgcggaatgatcatctcaacgtcag | Verification of *cpdA* cloning |
| 1. Ptet-ihfA-P1 | aatgtgtagaggcattaaaagagcgattccaggcatcattg agggattgaatgtgtaggctggagctgcttc | Construction of P*tet* driving *ihfA* at the *ihfA* locus |
| 1. Ptet-ihfA-P2 | cccaagcttatcaaacagatattctgacatttcagcttttgtaa gcgccatggtacctttctcctctttaatgaattc | Construction of P*tet* driving *ihfA* at the *ihfA* locus |
| 1. ihfA-ver-R | tcgtctttgggcgaagcgttttcgac | Verification of P*tet*-*ihfA* at the *ihfA* locus |
| 1. Ptet-ihfB-P1 | gtttcgtcctgtaatcaagcactaagggcggctacggccgcc cttaatcaatgtgtaggctggagctgcttc | Construction of P*tet* driving *ihfB* at the *ihfB* locus |
| 1. Ptet-ihfB-P2 | gaatgtgcgattgctgggtggcaagtctttctatcaattctgact tggtcatggtacctttctcctctttaatgaattc | Construction of P*tet* driving *ihfB* at the *ihfB* locus |
| 1. ihfB-ver-R | tccagttctactttatcgccagtcttc | Verification of P*tet*-*ihfB* at the *ihfB* locus |
| 1. PtetM2-Xho-F | atactcgagactctatcattgatagagtttg | Cloning P*tet*-*ihfA* and P*tet*-*ihfA*.G62E into pKDT |
| 1. ihfA-Bam-R | ataggatcctggtatccgttctgctgaagtgtcatg | Cloning P*tet*-*ihfA* and P*tet*-*ihfA*.G62E into pKDT |
| 1. ihfA-R | cgttttcgggttacgttccgggcgttgattcttatc | Construction of *ihfA*G62E |
| 1. ihfA-F | gaatcaacgcccggaacgtaacccgaaaacg | Construction of *ihfA*G62E |
| 1. intS-P1 | agatttacagttcgtcatggttcgcttcagatcgttgacagccg cactccatgtgtaggctggagctgcttc | Construction of P*tet*-*ihfA* and P*tet*-*ihfA* G62E at the *intS* locus |
| 1. ihfA2-P2 | agttgttaaggtcgctcactccaccttctcatcaagccagtcc gcccaccattactcgtctttgggcgaagcgttttc | Construction of P*tet*-*ihfA* and P*tet*-*ihfA* G62E at the *intS* locus |
| 1. intS-ver-R2 | aaaggaatgaagtctatcatccaagtc | Verification of P*tet*-*ihfA* and P*tet*-*ihfA* G62E at the *intS* locus |
| 1. PIS1-Xh-F | ttactcgaggtgatgctgccaacttactgatttagtgtatg | Cloning IS1 transposase promoter into pKDT |
| 1. PIS1-Bm-R | ataggatccatccaacgccattcatggccatatc | Cloning IS1 transposase promoter into pKDT |
| 1. PIS5a-Xh-F | atactcgaggaaggtgcgaacaagtccctgatatgag | Cloning Ins5A promoter into pKDT |
| 1. PIS5a-Bm-R | aatggatccaatgctgcatgcagtgaatgcgtagc | Cloning Ins5A promoter into pKDT |
| 1. PIS5b-Xh-F | atactcgaggaaggtgcgaataagcggggaaattcttc | Cloning Ins5CB operon promoter into pKDT |
| 1. PIS5b-Bm-R | attggatccatttcgcatcatcaagcgacagttc | Cloning Ins5CB operon promoter into pKDT |
| 1. PIS-Z-P1 | agccgcgctttcttcttcgcgacgttcgttaacaacaacttcta atttttcatcaaagggaaaactgtccatatgc | Construction of IS1 and IS5 promoter *lacZ* reporter at the *lac* locus |
| 1. PIS1-Z-P2 | gtaaaacgacggccagtgaatccgtaatcatggtcatagct gtttcctgtgtgttaggagggacagctgatagaaacagaag | Construction of IS1 transposase promoter *lacZ* reporter at the *lac* locus |
| 1. PIS5a-Z-P2 | gtaaaacgacggccagtgaatccgtaatcatggtcatagct gtttcctgtgtgttattcactgtcggcgaaggtaagttgatg | Construction of Ins5A promoter *lacZ* reporter at the *lac* locus |
| 1. PIS5b-Z-P2 | gtaaaacgacggccagtgaatccgtaatcatggtcatagct gtttcctgtgtgttacgataaccaactggcgatgttattcac | Construction of Ins5CB operon promoter *lacZ* reporter at the *lac* locus |
